# EMDR as Treatment Option for Conditions Other Than PTSD: A Systematic Review

**DOI:** 10.3389/fpsyg.2021.644369

**Published:** 2021-09-20

**Authors:** Charles Scelles, Luis Carlo Bulnes

**Affiliations:** ^1^Adult Psychiatry Department, Université Catholique de Louvain – Saint Luc University Hospital, Brussels, Belgium; ^2^La Métairie Clinic, Nyon, Switzerland; ^3^Adult Psychiatry Department, Geneva University Hospital, Geneva, Switzerland; ^4^Brain, Body and Cognition Research Group, Vrije Universiteit Brussel, Brussels, Belgium

**Keywords:** mental health, systematic review, trauma, EMDR, eye movements desensitisation and reprocessing

## Abstract

Eye Movement Desensitisation and Reprocessing (EMDR) is a treatment for post-traumatic stress disorder (PTSD). The technique is known to facilitate reprocessing of maladaptive memories that are thought to be central to this pathology. Here we investigate if EMDR therapy can be used in other conditions. We conducted a systematic literature search on PubMed, ScienceDirect, Scopus, and Web of Science. We searched for published empirical findings on EMDR, excluding those centred on trauma and PTSD, published up to 2020. The results were classified by psychiatric categories. Ninety articles met our research criteria. A positive effect was reported in numerous pathological situations, namely in addictions, somatoform disorders, sexual dysfunction, eating disorders, disorders of adult personality, mood disorders, reaction to severe stress, anxiety disorders, performance anxiety, Obsessive-Compulsive Disorder (OCD), pain, neurodegenerative disorders, mental disorders of childhood and adolescence, and sleep. Some studies reported that EMDR was successful in usually uncooperative (e.g., Dementia) or unproductive cases (e.g., aphasia). Moreover, in some severe medical conditions, when psychological distress was an obstacle, EMDR allowed the continuation of treatment-as-usual. Furthermore, the effects observed in non-pathological situations invite for translational research. Despite a generally positive outlook of EMDR as an alternative treatment option, more methodologically rigorous studies are needed. We discuss the advantages and limitations and possible implications for the hypothesised mechanisms of action.

## Introduction

Eye-Movement Desensitisation and Reprocessing (EMDR) is a psychotherapeutic approach, initially destined for the treatment of Post-Traumatic Stress Disorder (PTSD) (Shapiro, 1989). During a therapeutic session, patients with PTSD had to perform a series of eye movement alternations sequentially and at different times. The symptoms subsided. The technique is articulated around several clinically relevant practises and procedures, such as relaxation techniques, installation and bolstering of inner resources, and training to face internal difficulties. EMDR follows an eight-phase protocol (see Table 1).

**Table 1:** Eight-phase protocol of EMDR according to Shapiro (1989, 2001, 2007).

**Phase**	**Action**
1	History and treatment plan
2	Introduction to EMDR protocol, and development of coping strategies
3	Evaluation of the treatment targets
4	Desensitisation and reprocessing
5	Incorporation of positive cognitions
6	Body scan (and reprocess of any remaining bodily negative sensation)
7	Relaxation (re-establish emotional stability if distress has been experienced and for use between sessions)
8	Re-evaluation

The therapist decides when is the right moment to proceed from one phase to another and is, therefore, able to decide to deepen a certain phase when judged necessary. During the desensitisation phase, the patient is asked to attend simultaneously to the chosen target (e.g., a traumatic memory) and to an alternative visual, auditory, or tactile bilateral stimulus (BLS). He is then asked to take a deep and slow breath and to briefly report whatever comes up, mostly sensations, memories or thoughts (Shapiro, 2001). BLS-sets, followed by these spontaneous associations, are repeated until the discomfort associated with the memory dissipates. The therapy can therefore vary, from the target that is chosen (e.g., a memory, a sensation, a feared situation), to the lengths or type of BLS applied, or the interventions that are made by the therapist between the sets (Tarquinio et al., 2017).

The therapy follows the Adaptative Information Processing Model (AIP) (Shapiro, 1994, 2001, 2007). This model postulates that the brain possesses an innate information processing system that assimilates new experiences by storing them into memory networks. These networks will therefore constitute the perceptions, negative beliefs, affects, and body sensations linked to the initial event. For instance, information that is inadequately processed in traumatic situations leads to the emergence of manifestations observable in post-traumatic stress. Bilateral Stimulation is supposed to facilitate the reprocessing of the maladapted information.

The underlying mechanisms of action are a topic of ongoing scientific debate. Theories are heterogeneous and broadly fall into three categories: Psychological, Psychophysiological, and Neurobiological. We briefly outline the main tenets of each (for a review, see Landin-Romero et al., 2018).

The first psychological model is based on the Orienting Response (OR) phenomenon, described by Pavlov in 1927. In the face of threat or novelty, a set of behavioural and physiological changes prepare to respond to danger. If no real threat is experienced, a relaxation response takes place. According to some authors, this relaxation response can desensitise traumatic memories by suppressing its associated disturbance. The dual attention task during the BLS seemingly activates the OR reflex (Armstrong and Vaughan, 1996) creating a “dearousal” effect, by which changes between sympathetic and parasympathetic responses should happen, triggered by an attention attracting stimulus (Söndergaard and Elofsson, 2008). However, the psychophysiological underpinnings of these responses have not been fully supported in the past (Söndergaard and Elofsson, 2008).

A second psychological model focuses on the Working Memory account, in which the crucial element is the competition between the vividness of traumatic memories and the BLS due to limited-capacity working memory resources. The competition leads to a decrease in the vividness of the treatment target (Andrade et al., 1997). However, demanding tasks during recall has been shown to reduce the vividness of emotional memories (Engelhard et al., 2010; de Jongh et al., 2013).

Psychophysiological approaches focus on the ability of BLS-sets to trigger specific changes associated with desensitisation. These changes occur as an increase of autonomic activity, coupled with an increase in respiratory rate during BLS, thus excluding the effect of a mere relaxation-response (Elofsson et al., 2008; Schubert et al., 2011, 2016). Conversely, a distinct psychophysiological model hypothesises that ocular BLS induces a REM-Sleep-like brain state. Because REM is characterised by burts of eye Movements, reduction of temperature regulation, alpha waves on EEG and activation within eye nuclei in the brain (Söndergaard and Elofsson, 2008), BLS seems to fit similar activity patterns. Because REM bolsters memory consolidation *via* the integration of emotionally charged autobiographical memories into general semantic networks (Born et al., 2006), BLS may help process memories similarly as well.

Finally, the neurobiological accounts have focused on changes and activation in the thalamocortical connexions (Llinas et al., 2002) and in the mediodorsal thalamus-Superior Colliculus-Amygdala circuits (Bergmann, 2008; Baek et al., 2019). Bilateral sensory stimulation has been shown to promote the attenuation of fear *via* a pathway mediated exclusively by the superior colliculus. Sustained stimulation of this pathway was necessary and sufficient to prevent reversal of fear extinction (Baek et al., 2019). The general view of these accounts is that BLS facilitates associative and episodic memory processing and retrieval crucially attenuating traumatic memories. In concert with an extinction-related response, the Reciprocal Inhibition Hypothesis, based on the incompatibility between anxiety and parasympathetic states (e.g., relaxation, feeding, sexual states), postulates that induction of a parasympathetic state, should induce extinction of anxiety. As such, eye movements are thought to induce such states (Söndergaard and Elofsson, 2008).

The clinical efficiency of EMDR in trauma-related disorders has been demonstrated in numerous meta-analysis (Davidson and Parker, 2001; Seidler and Wagner, 2006; Chen et al., 2015; Moreno-Alcázar et al., 2017), leading to its recommendation as a first-line treatment for PTSD by the World Health Organization (2013). However, it has yet not been included in therapeutical guidelines for any other medical conditions, certainly due to the sparsity of studies of EMDR outside PTSD. Still, the techniques' official training manuals as well as EMDR textbooks (Tarquinio et al., 2017), describe possible adaptations of the protocol to treat conditions such as addiction, distress reaction to severe medical conditions, depression, and anxiety disorders. Moreover, the recent evolution in the definition of trauma and its causes, as described in the DSM diagnostic manual (North et al., 2016), the criticism of the categorical approach as diagnostic criteria (Vanheule et al., 2019), and the implication of distressing life events in numerous psychiatric conditions (Kim and Lee, 2016; Copeland et al., 2018, Overstreet et al., 2017), make the efficiency of EMDR outside PTSD plausible. It would indeed be surprising for a therapeutic tool to be only effective in treating one specific disorder. We, therefore, performed this systematic review, aimed at investigating the evidence of the use and efficacy of EMDR in other conditions than PTSD by using the PRISMA guidelines for transparent reporting of reviews and meta-analyses. A previous similar work (Valiente-Gómez et al., 2017) searched for this in specific conditions, and only included RCTs.

We further sought to integrate all types of studies, including case studies. Indeed, RCTs cannot be considered as the only valid scientific level of proof (Anglemyer et al., 2014). Including all types of studies allows a broader overview, which seems necessary given the urgency of knowledge in a context where psychiatric disorders are still one of the deadliest medical conditions (Walker et al., 2015).

## Methods

### Database Search and Filtering

**Figure 1:**
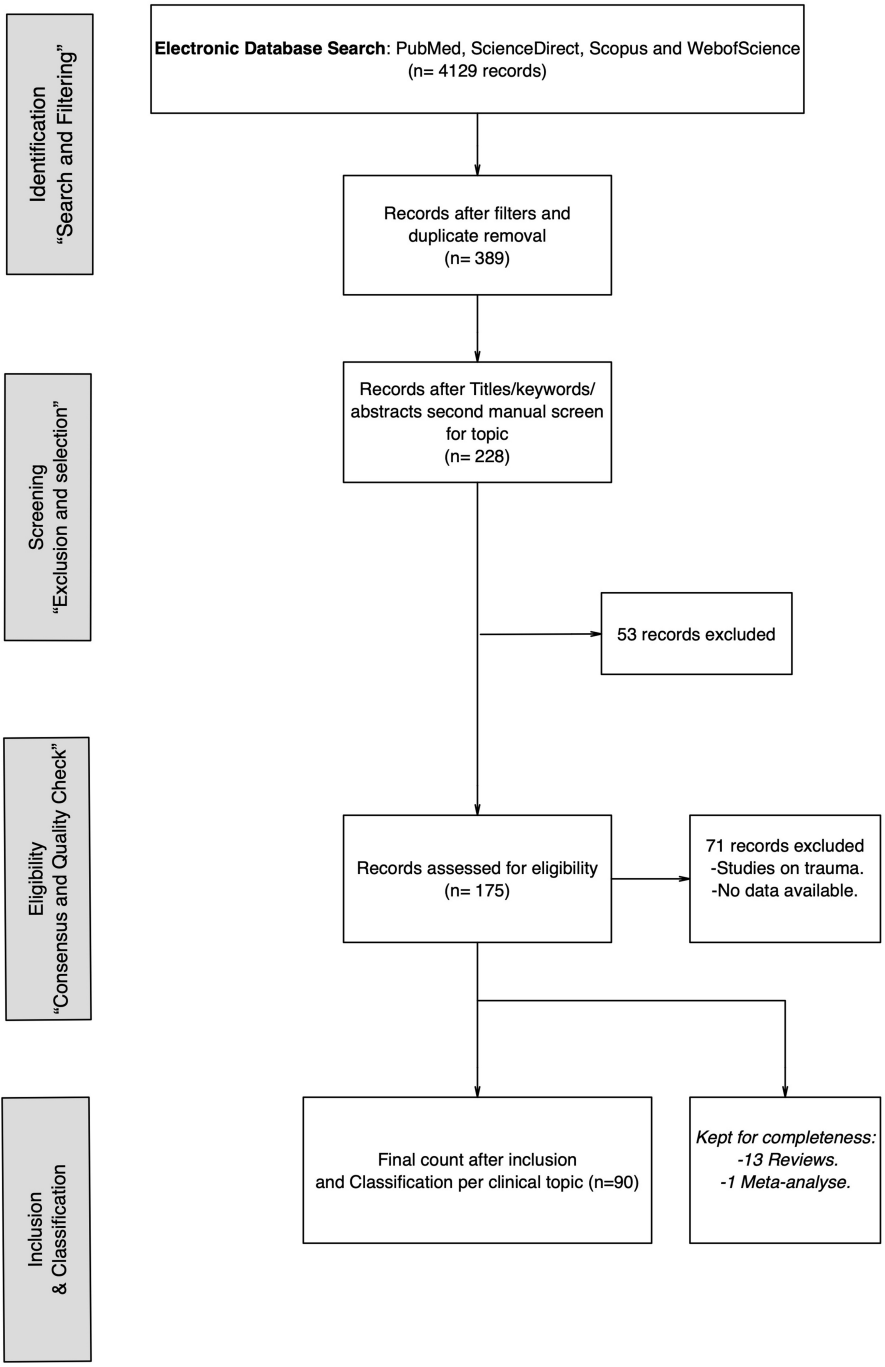
Flowchart depicting the search, selection, and inclusion of the items of the systematic review.

We conducted a systematic literature search among PubMed, ScienceDirect, Scopus, and WebofScience similarly to those previously used in systematic reviews related to Trauma and PTSD (Valiente-Gómez et al., 2017; Bloomfield et al., 2020). The studies included published works up to 2020 and are reported following the Preferred Reporting Items for Systematic Reviews and Meta-Analysis Guidelines (PRISMA) (Figure 1).

The first search was performed automatically by title within each database, and included the keywords “EMDR,” “Eye-Movement Desensitisation and reprocessing” with the Boolean operator “OR” (e.g., “EMDR” OR “Eye-movement desensitisation and reprocessing”). Because this review aims at reporting on the use of EMDR procedure in cases not directly related to PTSD, all searches within the databases were automatically filtered excluding titles that included the terms “PTSD,” “Post-traumatic stress,” “Posttraumatic stress” defined with Boolean operators “AND” and “OR” (e.g., exclusion of titles with the terms “EMDR” AND “Post-traumatic stress).”

Note that we decided not to use the term “disorder” because many articles used the variant “post-traumatic stress symptoms,” therefore reducing to post-traumatic stress was more efficient.

The resulting list of articles of all four databases was imported into Mendeley Desktop. A cheque for duplicates was performed, and duplicate articles discarded accordingly.

A second search was performed within the remaining records based on the exclusion topics (i.e., PTSD, Post-traumatic stress, posttraumatic stress) searched first by title, then by Keywords and finally within each abstract. We manually discarded those articles that were specific to the study of PTSD and trauma.

### Article Selection

Articles were further scrutinised following different criteria: (1) Articles were in full and constituted empirical findings (2) Articles were either Group studies or Case studies. Both instances of studies are reported. Conference proceedings, Theory or Opinion articles were excluded. Review articles were included for completeness, although the review articles are more general in a given topic (e.g., pain) and did not focus on EMDR outside trauma *per se*. Note that the final selection led to the systematic exclusion of articles that mentioned that the subjects had been exposed to trauma. Therefore, articles included had to focus on the treatment of disorders that could not be assimilated to post-traumatic stress or traumatic events (e.g., acute stress disorder, violence, grief).”

All articles were selected by both authors independently, and selections later interchanged for comparisons. Final inclusion was achieved after mutual consensus. One of the authors (C.S) is an EMDR trained psychiatrist.

### Article Classification

Remaining articles were classified in different groups following the ICD classification system (International Classification of Diseases and Related Health Problems) with three exceptions: Sleep disorders, Mental disorders of childhood and adolescence and non-psychiatric conditions (e.g., Neurodegenerative disorders).

## Results

The results of the present review were organised in five main sections: (1) General search results and classifications, (2) General Studies Characteristics, (3) Overall EMDR reported success, (4) Quality of evidence assessment of group studies, and (5) Main findings per category. Because results and studies are heterogenous, a meta-analysis was not considered and only a descriptive approach was followed.

### General Search Results and Classification

The database search resulted in a total of 4,129 records. The original number was reduced to 389 records after philtres and duplicate removal. Duplicate removal was performed manually. After the second manual and independent screening, records focusing on trauma or PTSD were removed based on specific title, keyword, and abstract inspection resulting in 228 records. A second round of inspection, independently by both authors resulted in further 53 records excluded. A total count of 175 records were assessed for eligibility, with a total of 71 records excluded based on the content. Papers were focused on the study of trauma specifically or data was not available (e.g., conference abstracts, full documents not found). From the final count of 104 papers, 90 were included as eligible research papers for review. Thirteen reviews and one meta-analysis were also included but will not be discussed as these included studies focusing on trauma and are mentioned only for completeness.

Of the 90 final studies, 14 different categories of clinical disorders were identified following the ICD classification system with the exception of sleep disorders, Mental disorders of childhood and adolescence and non-psychiatric conditions (Table 2). See supplementary material for a general overview of all the studies included in this review and their summarised characteristics (Supplementary Tables S1–S14).

**Table 2:** Summary of the article classification and the total of sample size of studies eligible for the systematic review.

**Article categories**	**Number of eligible studies**
Mood disorders	10
Reaction to severe stress	7
Anxiety disorders	11
Performance anxiety	8
Obsessive-compulsive disorder	7
Pain	22
Somatoform disorders	4
Sexual disorders	2
Addiction	5
Eating disorders	1
Disorders of adult personality	4
Neurodegenerative disorders	2
Sleep	1
Mental disorders of childhood and adolescence	6

### General Studies Characteristics

#### Design

The studies could be distinguished by several features. For instance, the type of study design. Only 1/3 of the total amount of studies constituted RCT's (*n* = 27), 24% corresponded to group-controlled (*n* = 10) or group studies (*n* = 12) and nearly half (46%) corresponded to case studies, case reports and case series (*n* = 41).

#### Protocol

The studies could further be distinguished by variations of the EMDR protocol reported. In some instances, the specific protocol was not specified, in which case it was considered as “unspecified.” Twenty-two different protocols could be identified, most were variations of the standard protocol described by Shapiro (2001), some integrated protocols where the standard protocol was used in tandem with other techniques or variations corresponded to the type of targets. Moreover, results demonstrated that some protocols were used also in different disorder categories (e.g., Phobia protocol used in Anxiety and OCD) and that in other instances, several different types of protocols were utilised within one single disorder category (e.g., in the pain category, at least six different protocol variations were reported). Furthermore, results showed that the standard protocol was reported as used in all disorder categories, except in somatoform disorders, Addictions and eating disorders for which we did not find explicit data of the protocol used. This suggests that there is not one specific protocol type per disorder category, although the standard protocol remains the most widely used. Crucially, some protocols devised for specific disorders may also be used in other types of disorders as well. See Supplementary Tables S15–S17 in supplementary material for an overview of the distribution of protocol variations across disorders categories and a distribution of studies by disorder category and intervention protocol.

#### Session Number and Time

Although the number of EMDR sessions was variable (between a single session to 72), it was possible to observe that the overall average number of EMDR sessions is of about 7–8 sessions (M = 7.61, SD = 2.11) regardless of the type of study (i.e., RTC, group-controlled, group study, case report, case study or case series). To estimate this, the specific reported average of number of EMDR sessions was taken into account, when this was not possible, the average of the min-max range of the reported number was included (e.g., “between 1 and 24”).

In regard to the time of each EMDR session, it was observed that on average, each EMDR session lasted about 78 min (SD = 8 min), ranging between 70 and 88 min, with the lowest mean session duration reported by the RCTs group of studies (Table 3).

**Table 3:** Summary of the distribution of session number, duration, and average sample size per study type.

**Study type**	**Sessions number**	**Session duration**	**Sample size**
RCT	7.6 (1–24)	70.6 (38–90)	26
Control group	6.24 (1–12)	88 (60–150)	20
Group study	6 (1–10)	80 (60–90)	14
Case study/reports	10.6 (1–72)/9 (1–32)	74 (60–90)	2

*The Min–Max reported range of all studies per group is shown*.

### Overall EMDR Reported Success

#### Reported Success

Overall, results demonstrated that EMDR resulted un positive outcomes in most cases, showing either improvements over time, or better results in comparison to a control group.

We operationalised success according to (1) Success (reported positive outcome), (2) Intermediate success (reported positive outcome) but either not better than the comparison group or comparison treatment or no treatment (e.g., not better than CBT), or only partial improvement, and (3) No success (reported lack of positive outcome or failure of treatment). We identified 76 studies reporting clear successful effects of EMDR, nine studies showing an intermediate success and five studies reported no positive effects or no improvement after treatment.

From the total count of studies that reported positive outcomes, over half (58%) of the amount corresponded to studies related to Pain, Anxiety, Mood, Stress. This suggest that the literature about the use of EMDR outside trauma that reported successful outcomes was higher in these categories. When considering the proportion of studies per category (e.g., total count of positive outcomes/ total number of studies per category), all categories showed high percentage of studies with positive outcomes (>75%), with the exception of addictions (60%; 3 out of 5) and OCD (57%; 4 out of 7). Importantly, in some categories only one or two studies were found (i.e., Sleep, Sexual disorders, eating disorders, neurodegenerative disorders), therefore these results must be interpreted with caution. See Supplementary Table S18 in supplementary material for an overview of reported success per disorder category and study type.

To better understand the results related to intermediate findings and those studies that reported no-improvements, we briefly summarise these.

Nine studies reported a positive effect, although the effects were not distinguishable from the effects of a comparison group or other treatment. Two RCTs studies on depression did not find that EMDR was different or better than CBT (Ostacoli et al., 2018; Minelli et al., 2019). It is important to note that the study by Ostacoli et al. (2018) was the only study included in mood disorders that used a different protocol than the standard protocol (i.e., DeprEnd). One group study on addictions reported that, out of eight participants included, three recovered during baseline period, two did not respond to treatment, and three improved during EMDR (van Minnen et al., 2020). Moreover, one group-controlled study on anxiety also found that, although there was an improvement in symptoms related to panic disorders, EMDR was not better than CBT (Faretta, 2012). Similarly, an RCT on phobia by Goldstein et al. (2000) found that EMDR resulted in significant reduction of symptoms of panic disorder with agoraphobia, as compared to a waiting list, but no difference was found compared to an attention-placebo control group. In the same vein, a controlled group study on performance anxiety by Brooker (2018) reported that EMDR was equally effective to hypnotherapy in reducing state-anxiety in musicians, with only slightly stronger statistical effects. Similarly, a controlled group by Muris et al. (1998) reported that only exposure *in vivo* alone was significantly effective in reducing fear and avoidance self-reports on spiders in children with spider phobia. The effects of EMDR were positive, although only as an additive effect to exposure *in vivo*. Finally, Böhm and Voderholzer (2010) as well as Mazzoni et al. (2017) described three patients each, with OCD treated with a combination of EMDR and ERP. In both studies, all patients improved in their symptoms at post-treatment, however, it was not possible to determine what can be accounted for by the EMDR procedure specifically.

Of the studies with no improvement outcomes, four were randomised controlled trials (Carrigan and Levis, 1999; Ray and Page, 2002; Marsden et al., 2018; Markus et al., 2020) and one was a case report (Brennstuhl et al., 2017b). In the study by Markus et al. (2020) on addiction, results not only did not show significant additive effects of AF-EMDR on TAU in drinking behaviour, but also reported that individuals in the control group had a more positive outcome than those with EMDR. In the study by Marsden et al. (2018) on OCD, no significant differences between treatments at post-test were found and only 30% of patients improved in OCD symptoms. Similarly, Ray and Page (2002) reported that EMDR did not result in statistically significant improvement of pain scores, only a group receiving hypnosis statistically improved symptoms. One RCT study related to performance anxiety had previously reported that EMDR treatment reduced SUDs of discomfort of fearful imagery, as well as physiological anxiety (SCL), but did not reduce public speaking anxiety and was not different or better than any of the other control treatments (Carrigan and Levis, 1999). Finally, a case study by Brennstuhl et al. (2017b) reported that EMDR did not have any effects on patients with chronic pain, although they found a tendency to decrease in scores in all measures.

This suggests that almost 26% of all the RCT's included (*n* = 27) in the review either did not find that EMDR was better than a control condition or did not find any significant effect to the treatment [23% if all group studies are taken into account (*n* = 49)].

#### Treatment Dropout/Adverse Events

To gauge the treatment drop-out and adverse events, we focused on RCTs, the group-controlled and the group studies (*n* = 49). In total, 14 studies reported on patient drop-out. Eight RCT (out of 27), Two control-groups (out of 10) and three (out of 12) group studies reported that patients had stopped the EMDR sessions before completion of treatment. In particular, high levels of drop-out in a single study were reported in the treatment of addictions [number of dropouts, Addictions (*n* = 19; Markus et al., 2020)], OCD (*n* = 17; Nazari et al., 2011), and Pain (*n* = 12; Mazzola et al., 2009). Overall, the reasons for drop-out, when specified and explicitly related to the treatment, were usually because of how hard it is to reminisce past memories, or to EMDR being time-consuming (e.g., Markus et al., 2020).

#### Quality of Evidence Assessment of Group Studies

As an attempt to provide the overall *quality of evidence* of all group studies, we performed an *ad-hoc* assessment of the group studies' quality, with particular focus on the controlled-group studies. We proceeded by partially following the *ad-hoc* GRADE system (Atkins et al., 2004) that considers features of each study in a hierarchical manner (Petrisor and Bhandari, 2007). To do so, we considered several criteria, such as the study design, the methodological limitations (e.g., control for selection bias), the types of controls, the outcome measures (e.g., were there multiple baselines? Was the main outcome measured *via ad-hoc* measures or were there standardised measures utilised?), the consistency of results (e.g., results were maintained at follow-up; results or interventions were not heterogeneous), and the precision and success of results. The result allowed to have an overall appraisal of the quality of evidence (see Supplementary Material for details).

It was observed that although the level of quality by design is considered the highest within the RCT studies, 60% of controlled group studies also proceeded by randomisation. Therefore, around 40% of controlled group studies presented the limitations of selection bias. The quality of the control types (e.g., comparison against gold-standard treatment, waiting list or no treatment) was also limited within the group-controlled studies. Only 50% of studies compared against either CBT or Treatment as usual, and only 59% within the RCT's, thus showing that the type of control conditions was only of moderate or average quality within controlled groups.

Further scrutiny on the measures used for the main outcomes revealed that only five studies used *ad-hoc* measures instead of standardised psychometric tools. Given the nature of the procedure, all studies performed a baseline measurement; however, only six of the total count of group studies performed multiple baseline measurements.

Conversely, a higher proportion of group-controlled studies did perform follow-up assessment (70%), not being the case of RCT's, where only almost half (48%) of studies performed follow-up measures. In regard to the outcome, all group studies and controlled group studies reported success (see Supplementary Table S18). However, only 50% of controlled studies reported higher success than the control condition (70% in the RCTs). The subsequent quality assessment focused on heterogeneity in the results (e.g., patients still needed antidepressant medications after EMDR; Loss of control comparisons at follow-up, etc.), which was moderate in the case of controlled studies (40%), with higher consistency in the case of RCT's (25%, low inconsistency). Crucially, we also observed a moderate (30%) heterogeneity in the types of interventions (e.g., the same patient group receives mixed therapies at the same time; the control condition is heterogeneous).

Further inconsistency concerned the maintenance of effects at follow-up. Only three out of six controlled studies that reported/performed follow-up demonstrated maintenance of effects after the post-test. In the case of the group studies or the RCT's, they all reported maintenance of effects at follow up when reported.

Regarding the outcome data, while almost the totality of group studies reported success in both the critical and the secondary outcomes, only 70% of the controlled group studies reported on secondary outcomes measures, of which 5/7 reported success in both. Interestingly, the majority of the RCT's reported success in the critical outcome only. Finally, as previously noted, the group and group-controlled studies were highly under-sampled (<30; Central Limit Theory) and only about 44% of RCTs' included sample sizes per group of more than 30, thus not satisfying sufficient sample sizes in any of the groups, further reducing the quality of the evidence.

In sum, the evidence's overall quality was moderate, particularly in the case of the controlled group studies.

### Main Findings Per Category

In the next section, main findings of all studies for each category are presented.

#### Mood Disorders

Ten studies were found related to EMDR and mood disorders identified within three categories: (1) Depression, (2) specific aspects of depression, and (3) specific subtypes of depression (see Supplementary Table S1).

##### Depression

Six RCTs evaluated the effectiveness of EMDR in Depressive Disorders. Three compared EMDR to CBT, three EMDR to TAU. One study performed a multicentre RCT and found EMDR to be as effective as CBT in Recurrent Depressive Disorder (Ostacoli et al., 2018). However, the study showed that EMDR results appeared sooner during therapy. Another study (Hofmann et al., 2014) found significantly higher number of remissions at post-treatment in the adjunctive treatment of EMDR in comparison to two randomised groups of patients with Unipolar Primary Depression, one receiving EMDR + CBT, and one CBT only. Similar results were observed in a study (Minelli et al., 2019) comparing EMDR to CBT in Treatment-Resistant Depression, finding EMDR to be as effective as CBT during hospitalisation, and superior at follow-up. Among the studies comparing EMDR to TAU, a recent study found EMDR to be significantly more effective on Quality of Life in a group of 70 patients suffering from Major Depressive Disorder (Jahanfar et al., 2020). Two other studies similarly found good tolerance of the EMDR intervention compared to TAU, crucially they found EMDR particularly interesting because of the speed of its therapeutic action (Gauhar, 2016) and because the EMDR group had fewer relapses at 1-year follow-up (Hase et al., 2015).

##### Specific Aspects of Depression

We found two articles dealing with more specific aspects of depression. First, one RCT (Fereidouni et al., 2019) relating to the severity of suicidal thoughts in MDD. This study found a significant decrease in suicidal ideas in the EMDR group compared to a routine treatment without intervention. In the same vein, we found one case study that examined the effect of EMDR on depression in three patients with specific social, educational and professional deteriorations (Rosas Uribe et al., 2010). Longitudinal data of the study showed a positive effect on the subjects' mood, in particular there was a change on emotional-cognitive processing and conceptual organisation of emotional representations.

##### Specific Subtypes of Depression

We found two studies that concern specific subtypes of depression. One study described a clinical trial on 60 subjects with a history of myocardial infarction (MI) (Behnammoghadam et al., 2015). Depressive symptoms are frequent among post-myocardial infarction patients and may cause adverse effects on cardiac prognosis. Results demonstrated a significant reduction in depressive symptoms (BDI) after 4 months EMDR weekly sessions. The authors highlight that EMDR is “an effective, useful, efficient, economical, and non-invasive” method for treatment and reducing depression in patients with MI. In the same vein, a case report by Guina and Guina (2018) showed successful effects of EMDR treatment of depression in a patient suffering from aphasia, a condition hardly accessible to psychotherapy. Therapy improved depressive symptoms and aphasia.

In addition, we identified one review (Carletto et al., 2017) that analysed the results of RCTs evaluating EMDR in MDD, with some articles included in this section. The authors concluded that literature still lacks controlled studies and those currently found present several methodological flaws. Despite the limitations, EMDR seems to be a promising therapy to treat depression.

#### Reaction to Severe Stress

Seven studies were found related to severe stress. We identified studies related to (1) Cancer, and (2) Other issues related to severe stress (see Supplementary Table S2).

#### Cancer

Four studies were found that evaluate the effectiveness of EMDR in distress related to cancer. One group study (Borji et al., 2019) tested the effect of only two sessions of about 1 h of EMDR on perceived stress in patients with gastrointestinal cancer, compared to a control group of patients receiving routine care at home. Results demonstrated a statistically significant reduction of stress levels. Similarly, another group study (Szpringer et al., 2018) found comparable results in the intervention group on anxiety, depression, and food intake in 37 patients with Multiform Glioblastoma. Moreover, the Sense of Coherence (e.g., overall perception of well-being) increased in the EMDR group, as it decreased among the controls. Furthermore, we also found a case study (Dinapoli et al., 2019) of a man suffering from laryngeal carcinoma who experienced a high level of distress during his first contact with the thermoplastic mask necessary for radiotherapy. An association of psychiatric medication and three sessions of EMDR resulted in a decrease of distress that allowed to start radiotherapy with only a little delay. Finally, a case report (Trznadel and Grzybek, 2017) showed a significant decrease of distress in three sessions EMDR in a patient diagnosed with malignant neoplasm of the breast. Both, negative emotions and physical sensations decreased.

##### EMDR in Other Health Issues and Severe Stress

Similar positive results were found in three RCTs that reported a decrease in anxiety and depression scores in, patients requiring haemodialysis treatment (Rahimi et al., 2019), and in patients suffering from spinal cord injury (Hatefi et al., 2019). In the same vein, a group study (Zolghadr et al., 2019) found a decrease of anxiety scores in one single session of EMDR in a group of pregnant women with a history of stillbirth compared to a control group.

These results support the literature review by Shapiro (2014) that reported that EMDR is efficient in the treatment of the psychological or physiological effects of adverse life-experiences and has preventive and rehabilitative potentials for patients and their families.

#### Anxiety Disorders

Eleven studies were found related to anxiety disorders. We identified studies related to (1) Panic disorders, and (2) Phobia (see Supplementary Table S3).

##### Panic Disorder

A first case series (Goldstein and Feske, 1994) found an effect of EMDR after five sessions in seven subjects suffering from panic disorder, reporting a considerable decrease in the frequency of panic attacks, fear of experiencing a panic attack, general anxiety, and thoughts concerning the negative consequences of experiencing anxiety. An RCT by same author (Goldstein et al., 2000) found no statistical difference between control and experimental group in patients who have panic disorder with agoraphobia, concluding that “EMDR should not be the first-line treatment for this disorder.”

In the same vein, another RCT (Faretta, 2012) found that EMDR had similar effects to CBT after twelve sessions, although with faster progress in the EMDR group. Interestingly, a number of sessions superior to 10, with at least three sessions of preparation were also needed in two case studies (Fernandez and Faretta, 2007; Bhagwagar, 2016) before observing a therapeutic effect of EMDR in cases of panic disorder with agoraphobia. Finally, a more recent case study (Nicolas and Vautier, 2017) reported that one patient recovered from panic disorder after one single session of EMDR. Seemingly, EMDR specifically benefited the retrieval of non-identified memories that could then be targeted in therapy.

##### Phobia and Other Anxiety Disorders

Three case studies, in arachnophobia (Muris and Merckelbach, 1995), aviophobia (Lapsekili and Yelboga, 2014), and emetophobia (de Jongh, 2012), met our research criteria. All three reported a good tolerance and positive effect of EMDR in a maximum of four sessions, with remission of phobic behaviour maintained at follow up. When no behavioural change was observed, EMDR seems to have allowed the patient to beneficiate from exposure therapy. Furthermore, we also found a pilot-study in General Anxiety Disorder (Farima et al., 2015) and a case study in Social Anxiety Disorder (Sagaltici and Demirci, 2019), both similarly demonstrating positive effects of EMDR in terms of efficacy in reducing pathological worry symptoms and maintenance of results.

In addition, our review identified two reviews and one recent meta-analysis on EMDR in Anxiety Disorders. The meta-analysis of RCTs evaluated the effectiveness of EMDR in anxiety disorder, in a total of 17 trials with 647 patients (Yunitri et al., 2020). Results demonstrated that EMDR is successful in the reduction of anxiety, panic, phobia, and behavioural/somatic symptoms. Both reviews (Faretta and Leeds, 2017; Faretta and Farra, 2019) found that EMDR is an effective therapy for panic disorder although there is a clear need for more controlled studies. Furthermore, EMDR therapy effectiveness suggests that its efficacy may not be restricted to panic disorder in general but for other specific phobias as well.

#### Performance Anxiety

Our review identified seven group studies and one case series related to performance anxiety and EMDR. We could distinguish between studies related to (1) Public speaking, (2) Physical performance, (3) Learning, and (4) Test anxiety (see Supplementary Table S4).

For instance, the first RCT that we identified (Foley and Spates, 1995) described a study where college students suffering from public speaking followed either a standard EMDR protocol, an EMDR protocol with moving audio stimulus instead of eye movements, an EMDR protocol with the eyes resting on the hands, or a no-treatment control condition. Although all EMDR treatments were more beneficial compared to the no treatment group, they reported no difference between EMDR treatments, both, at post-test and follow-up. Crucially, the improvements were specific to subjective reports of communication anxiety but failed to improve subjective reports of public speaking anxiety measures and their behavioural assessment. Furthermore, no significant psychophysiological changes were reported. The results of this study suggest that the EMDR effects were related to subjective and general aspects of communication rather than specific fears. A similar study (Carrigan and Levis, 1999) assessed the effects of EMDR on fear and confidence reports of public speaking. Participants expressing fear of public speaking were assigned to four different protocols: (1) Eye movements and fear-relevant imagery, (2) Eye movement and relaxing imagery, (3) No Eye movement and fear-relevant imagery, and (4) No Eye movement and relaxing-imagery. The protocol focused on memories associated with fear of public speaking. The results specifically failed to reveal any substantial effect of eye movements on the outcome variables and only demonstrated that the subjective units of discomfort, as well as skin conductance values, were overall lower in the relaxing-imagery conditions. The authors suggested that the only beneficial effects of EMDR, may thus be merely related to the therapist-cued visualisations of fearful images, because exposure, even at an imaginal level, is beneficial. Thus, further supporting the view that exposure therapy may be a critical prophylactic to fear-related distress. Interestingly, a more recent study (Aslani et al., 2014) found opposing results. In this study, speech anxious students followed either a standard protocol or no treatment. However, the protocol did not focus on any memory specifically related to speech. Instead, participants were invited to imagine any disturbing situation to which subsequent eye movements followed. Results showed significant reductions of Confidence of speaker measures as well as speech anxiety at post-treatment in the EMDR group, but no difference observed in the control group. Interestingly, while this study suggests that EMDR may be beneficial for speech anxiety, it is difficult to gauge the specific benefits of eye movements to speech anxiety *per se*. Participants were tested on unspecific imagery; as such, it could be that they were simply more relaxed in general after the procedure and not actual “reprocessing” occurred.

In regard to physical performance and EMDR, three studies were found. One study (Rathschlag and Memmert, 2014) used an advanced version (Wingwave method) of EMDR to reduce anxiety in a group of non-pathological participants. This method combines standard EMDR and a muscle test (BDORT). Subjects were assigned to an EMDR group or a no-treatment control group. In the experimental group, participants were asked to self-generate any memory that they judged to be anxiogenic and were later assessed in their subjective reports of anxiety, and their physical performance in the pulling-force test (i.e., finger muscle contraction). A crucial element of this technique is that after EMDR if this technique is useful, there should be more muscle strength because of overall muscle relaxation allowing for enhanced control of movement. Their results demonstrated that all anxiety measures significantly decreased at post-treatment in the experimental group, and as expected, the mean strength rating for the finger pulling measurement was significantly increased. These results suggest that EMDR did have anxiolytic and relaxing effects. In the same vein, a clinical study on the effects of EMDR on musical performance (Brooker, 2018) demonstrated the effects of two sessions of either hypnotherapy or EMDR on objective performance and performance-anxiety within advanced pianists suffering from music performance anxiety. Both therapies were significantly effective in reducing state-anxiety from a short non-public baseline performance (2 min) to a post-treatment performance (2 min). However, a self-report assessment on perceived anxiety revealed that all treatments significantly reduced the participants' perception of anxiety immediately after the performance, with the EMDR group producing slightly stronger effects. Crucially, the assessment of their actual performance increased after hypnotherapy and EMDR, suggesting that both psychotherapies were similarly successful in reducing acute anxiety related to music performance. In the same vein, a case series (Falls et al., 2018) targeted prospective imagery on competitive golfers instead of focusing on past memories. Results demonstrated how anxiety and negative cognitions about future-oriented imagery were reduced with EMDR, further demonstrating a positive impact on performance as well.

Finally, we found two group studies related to testing anxiety and learning. One study (Maxfield and Melnyk, 2000) reported beneficial effects of EMDR on prospective test-related anxiety in test-anxious university students. In their study, one single session was sufficient to produce significant reductions in several measures of anxiety scores immediately after the EMDR treatment (vs Waitlist), with the treatment having lasting effects previous to examinations. More recently, another study (Vauthier et al., 2019) extended the EMDR beneficial findings to learning, specifically in mathematics, a well-known anxiogenic subject for university students required to pass a mandatory high-level mathematics contest. One single session of EMDR, focusing on past anxiogenic experiences related to learning mathematics revealed significant effects in reducing the negative-emotion bias related to mathematics in general. It resulted in an improvement of self-efficacy scores in mathematics at post-test. These results suggest that one-shot, short EMDR sessions are advantageous on anxiogenic experiences related to past as well as prospective memories.

#### Obsessive-Compulsive Disorder

We found seven studies related to Obsessive-Compulsive disorders (OCD). Five studies were case reports, and only two were group studies. It was possible to identify (1) studies reporting comparisons between CBT with exposure therapy (ERP) and EMDR, (2) studies comparing EMDR to medication, and (3) studies assessing variations of the standard EMDR protocol (see Supplementary Table S5).

Among the studies comparing CBT with exposure therapy and EMDR, an early report (Böhm and Voderholzer, 2010) described three patients with OCD diagnosis (e.g., obsessions of control, sexual and aggressive obsessions, washing habits) that in addition to exposure therapy (ERP) received EMDR in three different modalities (i.e., patient 1: EMDR first and then ERP, patient 2: ERP first and then EMDR, and patient 3: alternations of ERP and EMDR). Results demonstrated that all three patients presented a clear reduction in their obsessive-compulsive scores, and this was maintained at follow-up. Similar findings were reported in a case report (Mazzoni et al., 2017) with three patients presenting similar behaviours (e.g., washing obsession, fear of aggression, and aggressive obsessions), also presented with a combination of ERP therapy and EMDR. Results showed that all OCD symptom-scores were reduced irrespective of modality at post-treatment. Interestingly, dissociative scores were also modulated by the therapeutic protocol. Along with trauma, dissociative symptoms constitute underlying mechanisms of PTSD, and according to the authors this element could help elucidate the mechanisms by which EMDR help improve OCD symptoms; possibly because OCD and PSTD could share overlapping processes. Because these studies used combined protocols of either therapy, it is difficult to assess, however, the precise contribution of either technique specifically. More recently, however, another study (Marsden et al., 2018) shed some light on the benefits of combining EMDR and CBT therapy based on exposure and response prevention (ERP). In their RCT, two groups of patients with OCD were assigned either to an EMDR or a CBT. They reported no significant differences between the groups at post-treatment or 6-month follow-up, challenging the idea that exposure can make people feel worse.

From the studies comparing EMDR to medication, one group study (Nazari et al., 2011) compared standard EMDR to treatment with the antidepressant (SSRI) citalopram 20 mg for 12 weeks. Crucially they report a reduction of obsessive-compulsive symptom scores in both groups; however, the reduction of scores in the EMDR group was significantly higher than with citalopram. In the same vein, we found a case report (Corrigan and Jennett, 2004) of an OCD patient that was not responsive to CBT nor to a series of antidepressants (Fluoxetine, Paroxetine, Clomipramine and Amitriptyline), although he was under fluvoxamine (300 mg/d) at the time of referral. They report that the patient felt so well after only two sessions of EMDR that she decided to stop taking fluvoxamine, alas bringing back her depressive state. EMDR was re-established after her recovery and was finished in five sessions while fluvoxamine was reduced to 50 mg a day. Interestingly, the patient reported a complete relapse 9 months later after having ingested one single capsule of an herbal product containing 3 mg ephedra alkaloids, a catecholamine release stimulant. Although the authors do not comment on this issue, EMDR might act by mediating changes through this same pathway. Results thus suggest a cooperative effect of EMDR to medication.

Finally, our review identified two case studies using variations of the phobia EMDR protocol that included a “(mental)video playback,” where the crucial element is to address current obsessions, instead of past negative experiences For instance, Marr (2012) showed preliminary research on patients that have either not been responsive to CBT or were reluctant to CBT because their symptoms were exacerbated by exposure. Irrespective of the protocol used, all four patients presented a net decrease of the obsessive-compulsive scores at post-treatment and at follow up 4–6 months later. Similarly, using the same protocol, Marsden (2016) later reported three cases of female patients with similar improvement in all obsessive-compulsive measures.

#### Pain

Twenty-two studies were related to EMDR and pain management. To better assess these, we organised the studies based on the aetiology of the pain symptoms, namely: (1) Chronic pain, (2) Phantom pain, (3) Fibromyalgia, (4) Migraine, and (5) Acute pain/experimental (see Supplementary Table S6).

##### Chronic Pain

Seven studies were related to EMDR and chronic pain management. We identified five group studies. The first group study we could trace compared the effects of EMDR to hypnotic suggestion (Ray and Page, 2002). In this RCT, participants were randomly assigned either to an EMDR group or a hypnosis group. Perception of pain was assessed within each therapeutic session as well as between sessions (e.g., different times of the day, each day for seven days). Crucially, the authors report that although both types of treatment resulted in numerical declines of perceived pain, only the hypnotic group reached significant levels, suggesting that hypnosis was more useful in reducing pain than EMDR during the treatment session. In another study (Mazzola et al., 2009), a mixed sample of patients suffering from chronic pain took place on a 12 weekly 90-min session of an adapted EMDR protocol that focused on the pain sensations. Their results showed significant decreases in scores in all of the different health and mental health assessments (e.g., SF-36 functioning survey, Anxiety, Depressive symptoms, and pain perception), demonstrating an efficacious effect of EMDR, despite the heterogeneous aetiologies of their sample. Interestingly they reported that among all the personality assessments, the most prominent one was obsessive-compulsive symptoms, present mostly in the headache patients who were in the majority. A later group study (Brennstuhl et al., 2016) compared two different EMDR protocols [i.e., Standard protocol (Group 1), Pain protocol (Group 2)] to a control group that followed classical psychotherapy (Group 3) on multidimensional components of pain perception (e.g., sensorial, cognitive, behavioural, emotional, PCL checklist, pain perception). The standard protocol was the most effective of all, and this already after five sessions. Interestingly, although no statistical group differences were determined in the PCL-checklist, the control group got worse, while the two EMDR groups ameliorated in their subjective report of traumatic experiences. Despite the fact this study focused on pain and not trauma *per se*, the results shed some light on the fact that chronic pain may have an underlying aetiology specifically related to traumatic experiences, which are therefore best approached with the standard protocol. Furthermore, two group studies on chronic pain have tested the effects of EMDR in patients with rheumatoid arthritis, both in the adult and the young. In the adults' study (Ghanbari Nia and Behnammoghadam, 2018) the authors compared an EMDR protocol with guided imagery and a waitlist control group. Scores on pain perception were all significantly reduced both in the EMDR and the imagery groups; however, the EMDR decrease of pain severity was even lower, suggesting that the EMDR group rated significantly less pain than the guided and the waiting list groups. Similar positive results were reported in the juvenile group suffering from idiopathic arthritis (Höfel et al., 2018). Young patients suffering from this condition may sometimes be intolerant to methotrexate (MTX), a drug usually prescribed to treat arthritis. Here, the authors showed that EMDR followed in eight sessions over 2 weeks was sufficient to improve the intolerance symptoms as well as the reported Quality of Life of the sufferers. The authors point out that many negative feelings in this condition might be related to anticipation (e.g., suggestion) of the adverse effects of MTX. Therefore, anticipation and/or suggestion (see Ray and Page, 2002) seems key to understanding pain management.

Finally, we identified two case studies. The first one assessed chronic pain with a dedicated pain protocol (Brennstuhl et al., 2017b). In this study no significant results were found after five sessions with EMDR, although pain scores showed a tendency to decrease, in all measures (e.g., pain perception, sensory and emotional aspects of pain). The second case study used a mixture of standard/pain protocol (Grant and Threlfo, 2002). Here, however, all patients were relieved from pain and the positive effects were maintained after 2 months post-treatment. Crucially, results showed an improvement in the patients' negative construals, which the authors suggest may be related to (de)conditioning effects of EMDR.

##### Phantom Limb

Three group studies and one pilot study were related to phantom pain sensations. The first three concerned limb amputations. For instance, one study (De Roos et al., 2010) described a group of patients that underwent an EMDR treatment after amputation between 1 and 39 years before treatment. They administered a combined protocol (standard and pain) during an average of 6 sessions, showing that the overall effect of time of a decrease in pain intensity was significant, and this effect was maintained still 3 months later. In the same vein, another study (Sinici, 2016) demonstrated a significant improvement in several pain perception and mood scales, with a positive outlook still 3 months after treatment. Crucially, in their study, the EMDR protocol was applied right 1 week after amputation, suggesting that an early EMDR procedure is beneficial in the control of phantom pain. More recently, an RCT comparing EMDR vs. routine care of patients that had undergone amputation of lower limb (Rostaminejad et al., 2017), demonstrated that a standard EMDR intervention applied as early as 2 months post-amputation has significant long-lasting effects on phantom limb pain (PLP)of up to 24 months post-treatment. One pilot study (Brennstuhl et al., 2017a) extended the limb-related finding with patients having undergone mastectomies. Using a mixture of standard and pain protocol, the authors report that between 5 and 10 EMDR sessions were sufficient to significantly decrease the phantom breast sensations and pain of the patients, as well as improve their scores in anxiety and depression questionnaires with results stable of up to 6 months post-treatment. These authors suggest that EMDR may target not only pain perception alone but also many other non-specific memories, such as traumatic memories.

Complementary to these group studies, five case reports were found, all of which equally report on positive outcomes after EMDR. For instance, three studies report positive results of patients having suffered from upper and lower limb amputations (Willensky, 2006; Schneider et al., 2008; Flik and De Roos, 2010). Similarly, another study reported on two patients suffering from phantom breast symptoms (Brennstuhl et al., 2015). However, although both patients reported experiencing positive psychological impacts after EMDR (i.e., anxiety, depression), only one of them experienced a reduction in pain sensation. Finally, we found one case study of a patient with paraparesis after trauma of the spinal cord (Oledzka et al., 2016). Crucially, the patient had significant improvements after 6 weeks of EMDR, both, in mood scales and the perception of pain. These studies highlight the positive effects of EMDR in amputees' pain and mood perception, irrespective of the cause of amputation or its anatomical level.

##### Fibromyalgia

Our review identified only one group study and one case series report, on the effects of EMDR in the treatment of fibromyalgia. The group study tested the effects of EMDR on the fibromyalgia impact questionnaire, as well as on reports of fatigue, anxiety and depression (Friedberg, 2004). The authors also assessed thermal biofeedback and pain ratings. Their results indicated a decrease in all the measures after EMDR treatment, and these effects were still valid after 3 months follow up. The thermal biofeedback revealed an average increase in hand temperature right after treatment, which indexes a relaxing effect of the procedure, therefore suggesting a clear somatic and autonomic effect of EMDR. In the same vein, the case series reported the effects of EMDR in seven patients diagnosed with fibromyalgia (Kavakci et al., 2012). Their key finding was a specific decrease in tender points after treatment and the decrease in scores in different variables, including anxiety, depression and trauma-related ratings. Of interest, after treatment, six out of seven patients did not meet the FMS classification criteria, suggesting that EMDR not only helped to decrease symptoms but also was effective to the extent patients no longer met diagnostic criteria.

##### Migraine and Headache

Two group studies were related to migraine headaches. In one of the studies (Marcus, 2008), migraine patients were assigned to either a group that received an integrated EMDR procedure or to a standard care medication group. Participants took part in either treatment during the mild to a late stage of an acute migraine episode. The integrated EMDR consists in first starting the unique 1 h-session with diaphragmatic breathing and craniofacial compression, and in a second time within the same session submitting the patients to eye-movements in a figure of eight patterns in blocs of 30–90 s. Patients were assessed immediately 1 h after treatment in the EMDR group or about 4 h after taking medications. The authors demonstrated that 95% of the EMDR patients had reached a score of zero in the SPL scale after 1 h, and only 5% of the medicated group reached the zero SPL score of no pain in the same time. No other difference was found 24, 48 h, or 7 days after treatment between groups. Thus, suggesting that the integrated EMDR protocol helped reduce or eliminate migraine faster, in a short time window immediately after treatment. In the second group study (Konuk et al., 2011), patients suffering from chronic migraine followed an EMDR headache protocol that targets disturbing events related to the headache episodes. After an average of eight sessions of EMDR, there was a significant decrease in headache frequency, but no reduction of pain intensity. These effects lasted after 3 mo follow up. In sum, these studies highlight how EMDR may be useful in the treatment of migraine, either as a prophylactic procedure or a treatment for acute migraine episodes.

##### Acute Pain

We identified two group studies related to acute pain and EMDR. The first study (Hekmat et al., 1994) with an experimental design, tested the hypothesis that EMDR could modulate the pain threshold, pain tolerance and endurance after participants had received noxious stimulations with ice. Two groups received either only EMDR or EMDR and music in comparison to a no EMDR control. Both procedures were similarly effective in changing the pain threshold, and both were better than the control condition. Pain tolerance, however, resulted in being higher in the EMDR and Music group, suggesting that music has additive effects on EMDR. Note that eye movements were manipulated with a moving dot on a computer screen. The second group study (Maroufi et al., 2016) reported on the effects of EMDR on postoperative pain in adolescents undergoing surgery. In this study, patients admitted for emergency abdominal surgery were allocated either to an EMDR group or a Non-EMDR control group.

Right after surgery and as soon as they had recovered consciousness, the patients started baseline tests and subsequently had a one 60 min EMDR or No-EMDR session. Post-intervention results indicated a significant difference between groups, mostly due to a decrease in pain intensity scores for the EMDR group and an increase in pain intensity in the No-EMDR group, suggesting that EMDR administered at the earliest has immediate positive effects on pain perception, and no intervention allows an increase in pain instead. In sum, this study highlights how the EMDR can counter the encoding of a traumatic, painful experiences at the earliest onset.

In addition, our search identified three reviews related to EMDR and Chronic pain management (Tesarz et al., 2014; Tefft and Jordan, 2016; Wicking et al., 2017) and one review related to Phantom limb (Niraj and Niraj, 2014). Only one of these reviews (Wicking et al., 2017) focused on EMDR in chronic pain by patients who do not suffer from PTSD. Note that some of the studies we found on chronic pain, were included in previous reviews, although, our review differs in the sense that it focuses on studies not specifically studying trauma or PTSD *per se*, independently of whether the patients presented symptoms related to PTSD and of whether this was assessed or not. In general, all reviews in the matter converge in that EMDR is a safe and effective alternative to usual treatments or other psychotherapeutic approaches in the management of chronic pain.

#### Somatoform Disorders

Four studies were identified that were not related to specific psychiatric conditions. We identified studies on (1) Tinnitus, (2) somatic symptoms disorder and (3) conversion hysteria/dissociative symptoms (see Supplementary Table S7).

In the first study described in the literature testing EMDR in tinnitus (Rikkert et al., 2018), patients suffering from tinnitus followed a composite EMDR procedure where they had first to elaborate on disturbing memories (Tinnitus and past experiences) and focussed on tinnitus only at a later session. Results demonstrated that the distress related to tinnitus experience was significantly lower right after treatment compared to a waiting list. In the same vein, a more recent smaller study (Phillips et al., 2019) showed that a combination of standard and a specific Tinnitus EMDR protocols is effective in reducing the perceived extent of the handicap caused by tinnitus (THI) and depressive symptoms while showing no effects on anxiety. However, because participants were submitted to two EMDR protocols sequentially they were not all naïve to the EMDR procedure. As such, it is difficult to gauge the additive effects of an immediately previous EMDR protocol on tinnitus management.

Relative to somatic disorders, one group study (Demirci et al., 2017) compared the administration of Duloxetine vs. six weekly sessions of 90 min EMDR in patients diagnosed with Somatic Symptoms Disorder (SSD). Results showed that both groups significantly improved on all measures, however, the improvement was higher in the EMDR group compared to Duloxetine, suggesting that EMDR may be considered as a first-line treatment in SSD. Furthermore, one case study (Chemali and Meadows, 2004) related to dissociative symptoms reported the case of a patient suffering from 3 years of daily psychogenic seizures. After EMDR, the patient experienced fewer events shortly after the beginning of the treatment and was seizure-free after 18 months, with results maintained at 3-months follow-up.

Finally, one review was identified dealing with Functional Neurological Disorders (Cope et al., 2018), finding that EMDR was successful in treating symptoms in four out of a total of five patients that followed EMDR in three studies.

#### Sexual Disorders

We found two case reports on sexual disorders (see Supplementary Table S8). The first study described two cases of vaginismus successfully treated after only three sessions of EMDR (Torun, 2010). The results were maintained at 2 months follow-up. The second report (Gaboraud, 2018) highlights a case of paedophilia in which the introduction of EMDR after 8 years of psychotherapy successfully reduced the patients' paraphilias and was associated with subjective mood improvement.

#### Addiction

Five studies were found in total related to (1) Alcohol dependence, (2) substance abuse and (3) pathological gambling (see Supplementary Table S9).

A recent RCT tested the adjunction of EMDR in addition to TAU in a randomised group of adult patients with alcohol use disorder (Markus et al., 2020). The study failed to demonstrate any additive effects in the TAU+EMDR group; possibly due to the population tested being chronic poly-morbid patients with poor social support. Another group study (Hase et al., 2008) compared alcohol cravings measured with the Obsessive-Compulsive Drinking Scale (OCDS) in two non-randomised groups. One group received TAU and the experimental group received TAU+ two sessions EMDR. The therapy target was addiction memories. Results demonstrated a statistically significant effect on craving as on depressive symptoms, with the maintenance of that difference at 6 months follow-up. Authors note that targeting the addiction memory in EMDR did not lead to a destabilisation of patients.

Furthermore, one case study (Qurishi et al., 2017) on substance abuse found a successful treatment of a Gamma-HydroxyButyric Acid (GHB) addict with EMDR in a few weeks, with effects maintained after 6 months of follow-up.

Finally, we found one group study (van Minnen et al., 2020) and a case series (Bae et al., 2015) on pathological Gambling. Despite their small sample size, they both found a potentially positive effect of EMDR as a treatment in this condition, in particular with positive effects still observed at 6 months-follow-up (Bae et al., 2015). Overall, the treatment was well-tolerated, and its efficacy appeared in a short period of time.

In addition to these studies, two reviews were identified on addiction (Pilz et al., 2017; Markus and Hornsveld, 2019). Both found that EMDR has a high therapeutic potential in the field of addictions, although the conditions by which EMDR operates in addiction remains unclear.

#### Eating Disorders

Only one case study related to eating disorders could be identified (Yasar et al., 2019). This study applied a combination of EMDR and CBT in an intermingled manner (EMDR-CBT-EMDR) to two young adult patients suffering from restrictive food intake disorder. Results showed positive results on depressive and anxiety scales, but also on food habits, that were reported as back to normal after treatment (see Supplementary Table S10).

In addition, two reviews 20 years apart were identified. The first found EMDR to be ineffective and risky because it could trigger false traumatic memories and would only delay the use of proven-effective therapies (Hudson et al., 1997). Conversely, a more recent review (Balbo et al., 2017) found the approach beneficial as a complement to standard treatment.

#### Disorders of Adult Personality

Four case studies reported on EMDR and disorders of adult personality (see Supplementary Table S11).

A first case report documented a case of a female patient presenting symptoms of Borderline Personality Disorder (BPD), and major depression and anxiety (Brown and Shapiro, 2006). Results demonstrated a clinically significant effect after 20 sessions of EMDR during 6 months with improvements in BPD symptoms, overall functionality and affect.

Similarly, the same number of sessions were later used in another BPD case study (Safarabad et al., 2018) demonstrating positive effects on BPD symptoms, dissociation scores and affect. One case report on attachment disorder (Wesselmann and Potter, 2009), showed that patients suffering from relational and interpersonal functioning improved in their cognitions about their relationships. Crucially, the EMDR therapy helped the patients shift toward a more positive attachment status. The therapies, however, were highly spaced in time (over a year) and EMDR was coupled to talk therapy as well. As such, it remains difficult to gauge the exact additive effects of the procedure. Finally, we found a case report on a patient detained in a high-secured hospital that followed EMDR targeting triggers of the urge to self-injury (Annesley et al., 2019). Eighteen EMDR sessions were to reduce the urge to self-inf-jury to zero. The authors also reported a benefit on other mental health indicators, such as mood, thinking, sleep, concentration, memory, and the experiencing of flashbacks.

#### Neurodegenerative Disorders

We found two case studies related to neuropsychiatric disorders (see Supplementary Table S12).

One study (Amano and Toichi, 2015) reported on the reduction of erratic behaviour in the elder with dementia. Three patients diagnosed with dementia (vascular and/or AD) were administered an “on the spot” EMDR protocol. Because of memory and behavioural issues, the standard protocol was shortened, and the report came from hetero anamnesis. Crucially, the target of the disturbing episodes concerned the patients' erratic behaviour (i.e., wandering, screaming). For instance, past memories were assumed to be relived through the behavioural disturbances and were considered as the target of the desensitisation process. The beneficial effects of the procedure were still reported by their caregiver at 6 mo follow-up in various behavioural changes, such as less cursing, reduced restlessness, and more smiling. More recently, another study (Van Der Wielen et al., 2019) reported a patient diagnosed with AD at the early stages. The patient suffered from distressful memory flashbacks. Results demonstrated that one single session of EMDR was sufficient to reduce the distress related to the flashbacks, although there was no difference in measures of depression and anxiety 3 months after the intervention. Importantly her neuropsychological assessments had not changed between assessments and follow-up, suggesting that one single EMDR session had effectively helped the patient in her subjective experience of distressful perceptions and memories.

#### Sleep

Only one study related to sleep met our inclusion criteria (Nia et al., 2019). In this RCT, 75 subjects with rheumatoid arthritis experiencing insomnia were assessed on the insomnia severity index. The study showed that EMDR and Guided Imagery were significantly effective in reducing insomnia in those patients. However, the effects of EMDR were significantly better than those of guided imagery (see Supplementary Table S13).

#### Mental Disorders of Childhood and Adolescence

A total of six studies in children and adolescents were identified; two are already discussed within pain management for conceptual reasons. Among these, we could distinguish between studies on (1) anxiety disorders, (2) mood disorders, and (3) autoimmune disorders (see Supplementary Table S14).

For instance, two group studies report on EMDR in phobia. In the first study (De Jong et al., 1997) a small group of young girls were assessed for spider phobia and disgust before and after one single EMDR session. The post-treatment values of spider phobia and disgust had significantly decreased. Interestingly, results also showed a significant disgust bias in phobic children, as compared to the controls. Crucially, a disgust bias was found also in the parents of phobic children who found spiders more fearful in general, and at the same time their mothers found spiders more disgusting than the parents of the control group. The second study on phobia (Muris et al., 1998) reported a group study on spider phobia comparing EMDR to exposure *in vivo* and a computerised exposure control group. A second phase was added where all patients received one session of exposure *in vivo* to gauge the extent to which any of the conditions at phase one could potentiate the effects of exposure *in vivo*. Results demonstrated that only exposure *in vivo* had significant effects in reducing fear and avoidance self-reports of spiders (e.g., SPQ, -C, SAM., BAT). EMDR was only effective alone to reduce non-verbal reports of fear of spiders and had significant additive effects at phase 2 to exposure *in vivo*, only in measures of avoidance. These authors suggest that exposure *in vivo* remains the best protocol to treat spider phobia in children. It is worth noting, however, that both group studies included very small samples, their statistical inferences should thus be taken cautiously.

Further evidence related to anxiety disorders in the paediatric population comes from a recent group study (Mariani Wigley et al., 2019) reporting that one single EMDR session before invasive and painful surgical procedures had significant effects in preventing anxiety in children. Children following EMDR complementary to non-pharmacological treatment were significantly less anxious before the procedure. Note that a similar group study was later reported (Höfel et al., 2018) in young patients suffering arthritis and being intolerant to methotrexate. However, we discussed this study in the section for chronic pain for conceptual reasons. Another study on EMDR in post-operative pain in adolescents (Maroufi et al., 2016) was also discussed in the section for pain for the same reasons.

In support of previous findings on anxiety, we found one case study (Verkleij et al., 2017) reporting on an adolescent with difficult to control asthma with exacerbations exclusively related to the perception of shortness of breath (vs the cause of shortness of breath). Here, the adolescent patient followed a mixed protocol where she first received CBT therapy, and then EMDR only at the last two sessions. Analyses using structural break-point modelling and reliable change-index demonstrated that results on primary outcomes were significant compared to baseline at post-treatment and as a follow-up. Crucially, the level and the trend of asthma exacerbations over time was significantly reduced and there was an improvement in physical and social activities, physical complaints and anxiety in general. However, although the outcome measures seemed better during the EMDR protocol, the authors did not gauge if this was either an additive or a specific effect of EMDR.

Furthermore, our review identified one case study related to mood disorders in the adolescent (Bae et al., 2008). The study reported two cases of adolescents with major depressive disorder and no history of trauma that received between three and seven sessions of EMDR. Results demonstrated how depressive symptoms decreased to close to zero, with a remission maintained at follow-up after 2 months.

Finally, in one case study related to autoimmune disorders (Guido et al., 2019), EMDR was performed in a young boy (11 yo) with paediatric autoimmune neuropsychiatric disorder -associated with streptococcus (PANDAS). The patient also presented a series of motor and mood-related disorders (e.g., vocal tics, motor tics, irritability) comorbid with obsessive-compulsive traits. The EMDR protocol focused on unfavourable experiences and coping strategies improvement. Results showed that the severity of tics and the obsessive-compulsive behaviour had reduced after therapy, suggesting that EMDR was effective in reducing a series of motoric-related disturbances in a very young infant.

*Note that during the review process it was possible to identify three recent studies, one by Hafkemeijer et al. (2020) on EMDR and personality disorders, one by Matthijssen et al. (2019) on psychosis, and one by Dominguez et al. (2020) on EMDR and depression, not included in our literature search. In the RCT by Hafkemeijer et al. (2020) randomised controlled trial, the intervention group received 5 90-min sessions of EMDR and was compared to a waiting list group. Results showed a significant improvement on psychological symptoms, psychological distress, and personality functioning in the EMDR group. The differences were significant between groups and results were maintained at a 3-month follow-up. In the study by Matthijssen et al. (2019), patients suffering with auditory hallucinations performed an emotional memory recall task under three conditions: a visual taxing task (EMDR steps 1, 2, and 3, Dutch protocol), an auditory taxing task (counting) and a control task (eye fixation). Crucially, the authors reported that both active tasks were similarly effective in reducing SUDs scores of emotional memories. Finally, Dominguez et al. (2020) reported a randomised controlled trial with patients showing symptoms of depression and mostly meeting criteria for a major depressive episode. Fortynine participants were randomly assigned to either an EMDR group (3 sessions), an Assertiveness training group or Treatment as usual (TAU). Specifically, no significant differences among treatments were found at post treatment. However, the authors stressed that the likelihood of not meeting criteria for depression after the adjunct EMDR was higher in this group than those following assertiveness training or TAU.

## Discussion

The present review aimed to investigate the uses of EMDR outside trauma and PTSD.

To our knowledge, this is the first review on EMDR that integrates different clinical uses of this technique and overviews the extent of its effectiveness within and across several pathologies. To date, the scarce literature on the subject focused on RCTs' only, and just a few specific major topics. Here we present an extensive body of work on the uses of EMDR where good care was taken to identify studies not focused on the study of trauma and PTSD. We analysed state of the art broadly, identifying -in a clinically relevant fashion- a series of topics to better categorise the scope of application of EMDR. We observed that the technique is used in many other situations beyond the traditional guidelines, and the number of studies and research on EMDR has consequently grown in the last 10 years.

Ninety studies met our search criteria. Results demonstrated that EMDR is helpful in a series of conditions in which the study's primary purpose was not the treatment of trauma. The recent review included all types of designs, although only one-third of the studies were randomised controlled trials with a majority corresponding to case studies, suggesting that systematic studies on EMDR remain scarce.

We identified 14 different clinical disorders, and most studies consistently reported positive outcomes in all different disorders identified. However, the number of group studies, controlled-group studies or RCT's reporting either intermediate results (e.g., positive but no better than a control condition) or no results (i.e., failure of EMDR effects) corresponded to between 23 and 26% of the literature included. Importantly, although several of the group studies reported a significant improvement that was not better than the one of the comparison groups or the comparisons treatment, the positive effects of EMDR seemed to appear faster, as has been reported in previous reviews (cf. Pain results in a review by Faretta and Leeds, 2017; Wicking et al., 2017).

Evidence of successful outcomes was found across all disorders, although results were less evident in the cases of OCD and Addiction, for which almost only half of the studies reported clear positive results. Importantly, in some categories that reported successful results, the evidence was also too scarce (e.g., sleep, sexuality), thus not allowing for a generalisation of the effectiveness of EMDR in these categories. However, nearly half (58%) of the total number of studies included, and that reported positive outcomes, were related to Pain, Anxiety, Mood and Stress, suggesting that albeit the use of EMDR for different conditions is broad, the evidence remains higher only in these categories.

It was also possible to distinguish the use of EMDR according to several parameters related to the protocol. For instance, the literature on EMDR showed that at least 22 variations of the standard protocol exist in the literature included, suggesting a vast heterogeneity of the techniques' procedure across clinical uses. Interestingly, different protocols were reported within a single category (e.g., DeTur in Addictions and Adult personality). This suggests that there is not one specific protocol type per disorder category and that some protocols devised for specific disorders may be used in other conditions. Thus, results highlight that a common mechanism underlying the effects of EMDR may be at play across certain conditions. Moreover, the standard protocol was used in almost all conditions, except in somatoform disorders, sexual disorders, addiction and eating disorders. However, not many studies were found in these categories, and it is difficult to assess whether specific variations of the standard protocol are more common or more effective for a particular disorder, and more evidence is still needed in these domains.

In the same vein, results showed that the number of EMDR sessions was variable across studies, as may be expected in psychotherapy. However, it was possible to estimate the average number of EMDR sessions per treatment, ranging between 7 and 8 sessions, regardless of the type of study.

Similarly, the average time per session was estimated to range between 70 and 88 min. Both results suggest that the number of sessions, on average, tends not to be too short (e.g., <5) and that each session lasts almost 1 h 30 maximum but is always slightly more than 60 min.

Although timings, as well as protocols, both depend on the needs of the patients in the clinics, it is essential to have a quantification of the reported parameters to understand all factors that may play a role in the assessment of the effectiveness of the technique. This is of particular importance when considering randomised trials where a certain homogeneity of parameters is of particular relevance to make the proper statistical inferences.

Finally, the results of the review demonstrated that the number of the reported dropout within the group studies were in general low, with only three specific studies reporting high levels of dropout (addictions: Markus et al., 2020; OCD: Nazari et al., 2011; Pain: Mazzola et al., 2009). Interestingly in some instances, the reasons for dropout were related to external causes and not only intolerance to the EMDR procedure (e.g., work schedule incompatibility). Thus, suggesting that overall, the use of EMDR was well-tolerated.

### Strengths and Limitations

The present review demonstrates how EMDR has a series of beneficial aspects, although its limitations are intrinsically related to the procedure's implementation. For instance, the low dropout and the reported success suggests that EMDR is safe to use in several conditions. The estimated average of treatment and session time was convergent among studies, and the relatively short nature of the treatment may also contribute to the patient's compliance, so contributing to its effectiveness. However, it is important to note that most of the studies we could find reported successful findings; thus, the relative convergence of the reports may also be affected by a publication bias. The scarcity of literature may indeed be due to the exclusive report of positive findings, yet even the studies with a high level of proof (e.g., RCT's) had several flaws. For instance, these studies were in general relatively small (e.g., an average sample size of 26 patients), and some studies were not better than a control condition, despite showing positive outcomes. Nevertheless, most results (85/90) reported a positive outcome, although the literature in several disorder categories was under-represented (e.g., Eating disorders, Sexual disorders, neurodegenerative disorders, and sleep).

#### Implications for the Effectiveness of EMDR

It is critical to distinguish between clinically meaningful results and statistical comparisons between treatments. The present review showed a discrepancy in the literature related to the effects of EMDR in the remission of symptoms in patients and the technique's efficacy compared to other alternatives. In some cases, EMDR was not better than a control group or a control treatment (e.g., CBT). This suggests that the systematic integration of case studies, group studies, and RCTs in future reviews is warranted. As such, the use and efficacy of EMDR in different conditions should be understood broadly. This further invites at making the distinction between what is clinically relevant (e.g., symptoms improvements) and what is an empirical issue (e.g., assessing EMDR against other treatments). For instance, it is not enough to report the positive outcomes; the proper and exhaustive assessment of EMDRs' efficacy compared to other alternatives is needed through rigorous randomised trials with sufficient sample sizes.

Another limitation in this regard was the inclusion of very heterogeneous patients in RCT's or group studies (e.g., Chronic pain, Addiction). Several group studies included patients with all types of complaints or aetiologies in one single group, a factor that might affect the overall outcome and interpretation of their trials. This might also explain specific high dropout reports, as was the case in addictions (e.g., Markus et al., 2020). Therefore, the effects of EMDR in sub-categories of major disorders should be tested as well.

Timing parameters (e.g., session number) were also related to the effectiveness of EMDR. Although an average length of therapy could be estimated, in some disorders, the studies were very long (e.g., adult personality); in some other particular cases (e.g., migraine), EMDR was beneficial as an immediate prophylactic procedure when applied at the earliest. Moreover, another issue that was related to timing was the observation that in some cases, EMDR was compared to a waiting list, thus giving the patient more time with the therapist, an element that cannot be disregarded as the relational aspects of EMDR are essential in the development of the protocol and undoubtedly have an impact on the therapy. Therefore, the effects of time should be taken into account when assessing the efficacy of the technique and devising trial protocols.

Furthermore, an important finding was the effectiveness of EMDR as compared to other treatments. In some cases, no benefit of EMDR as compared to either exposure therapy (ERP) or CBT was found (e.g., OCD, Performance Anxiety). However, we found reports that EMDR was beneficial in reducing medication (e.g., citalopram and fluvoxamine) intake. Therefore, it is vital to gauge the effects of EMDR with several other alternatives.

Finally, in line with our findings, and as an addition to our quality assessment results, several recent meta-analyses show that, although EMDR is overall effective, the size of the effects remain small to moderate. Two meta-analyses on patients diagnosed with major depression (MDD) or presenting subclinical symptoms of MDD and other depressive disorders [Carletto et al., 2021 (*n* = 9); Dominguez et al., 2021 (*n* = 10)] reported that although the effects of EMDR for depression where large (Carletto et al., 2021), these were moderate compared to other treatments. However, EMDR is more likely to reduce depressive symptoms than CBT (Dominguez et al., 2021). More recently in a meta-analysis that considered only studies with patients formally diagnosed with MDD, Yan et al. (2021) (*n* = 8), EMDR was significantly more effective than CBT, further highlighting the importance of limiting the heterogeneity of patients in future RCT's. The authors point out the effects of low compliance in CBT (e.g., CBT is more time consuming) as a possible factor that bolsters EMDR superiority. Interestingly, a meta-analysis of studies of EMDR and anxiety by Yunitri et al. (2020) (*n* = 17), showed that time, duration, and recurrence of therapy do not modulate EMDR's effectiveness. Crucially, EMDR was effective in the treatment of anxiety, panic and phobia, although -surprisingly- they found no effects on traumatic feelings. Similarly, a subsample (*n* = 16) of a broader meta-analysis on EMDR and mental health by Cuijpers et al. (2020) (*n* = 76) corroborated positive effects of EMDR on anxiety disorders, although the effects of EMDR on PTSD were inconclusive, primarily because of large heterogeneity of studies included. Note that the high uncertainty of the evidence noted by Cuijpers et al. (2020) was further exemplified by the fact that analysis on a small subset of dismantling studies reported no difference of effect sizes between effects of partial and full EMDR protocols.

In sum, recent meta-analyses highlight several issues raised about the effectiveness of EMDR also present in this review. The heterogeneity of patients affects the outcomes. Despite the moderate evidence, EMDR clearly has clinical benefits, and meta-analytical findings particularly support the effectiveness of EMDR within anxiety and depression, with emphasis on the treatment of fear and the use of DeprEnd^®^ protocol, respectively.

The issue at hand regarding EMDR's effectiveness as compared to other therapies, may not solely be the question of whether EMDR is better (e.g., against an alternative), or what factors support its effectiveness (e.g., compliance) but also, the overarching implications such as the cost-effectiveness-benefit balance. Therefore, the economic and social impacts of EMDR should also be considered and studied further (Carletto et al., 2021).

#### Differences Between EMDR in Children and the Adult

Results were similar in both age groups, and overall, the majority of children's results were also related to pain, mood and anxiety. However, contrary to studies in the adult, we did find literature on phobia in children. The lack of studies on phobia in the adult may be related to the fact that phobia may usually be approached in the context of trauma in older patients and were thus not identified and included in the present review. Crucially, results showed that the parent-child correlation toward negative bias in phobia is very high. It is therefore crucial that, in tandem with the EMDR procedure, the parents of children with a phobia be also monitored to help reduce the possible risk of relapse, and suggests that in the adult with phobias, the context and immediate entourage should also be explored. In all, EMDR seems a safe procedure to use in the young, and literature showed how EMDR is successful, particularly as an anxiolytic procedure.

#### Implications for EMDR Possible Mechanisms of Action

Our review also showed that studies presented several overlapping factors. Among these, a central element was the nature of the targets, suggesting there is a possible common mechanism across disorders by which EMDR is operational. Almost all treatments reported were related to affective memory, regardless of whether this memory was related to past memories, remembered present or prospective memory. Indeed, affect is indissociable from memory systems, and the interplay between the triad of body-memory-affect is the cornerstone of affective consciousness itself (Panksepp, 2005; Vandekerckhove et al., 2014; Ledoux et al., 2017). EMDR seems specifically related to how these memories become disturbing.

Interestingly, the finding that EMDR can be successful in patients with frontal pathologies (i.e., neurodegenerative disorders) raise questions about the possible underlying mechanisms. During EMDR, the Prefrontal cortex (PFC) has significant involvement in the treatment of traumatic memories (Pagani et al., 2012) although few instances have shown opposite results (see Landin-Romero et al., 2018). Dementia is mainly related to disturbances of PFC (Burgmans et al., 2009). Therefore, these results are of interest to understand how EMDR operates, although the evidence was limited. Similarly, studies that support other neurobiological accounts remain scarce as well (e.g., sleep studies and similarities of EMDR with REM sleep). Moreover, some studies reported that EMDR is not better than a suggestion-focused therapy (i.e., hypnosis) in some cases for pain, so exposing an opportunity to understand the cognitive mechanisms of action of EMDR. As such, the results invite to elucidate the effectiveness of EMDR in relevant conditions (e.g., sleep, neurodegenerative disorders) and systematically compare to other techniques (e.g., CBT, Hypnosis).

#### The Omnipresence of Trauma in the Literature

The operational aspects of this psychotherapy depend on how the disturbing symptoms are conceptualised and contextualised. The therapists' approach to the patients' symptoms determines the target memories, and by doing so, the link between the symptom and the affective memory can be brought about. For this reason, to differentiate what is and what is not traumatic is a very delicate endeavour and leaves open the question of what precisely a target is and if the target memories of the therapy are not just a product of the therapists' desiderata. For instance, a significant limitation of this body of work remains the difficulty in the inclusion of research and studies not dealing with trauma, either as the primary target or as specific comorbidity. Almost all studies converged in the idea that negative memory representations and their affective component were the culprits of the negative symptoms treated by EMDR, most likely the product of traumatic experiences. For example, both studies on sexual disorders in their conclusions could only link the symptomatology to traumatic experiences. Thus, making difficult to assess the hypothesis that a particular disorder (e.g., sexual disorders) may also be related to non-traumatic memories and invites the question of whether it is indeed possible to understand EMDR effects and the underlying mechanisms outside the realm of trauma.

Together, our review suggests that despite the consistently reported success of EMDR in the treatment of several disorders, clearly more rigorous research is needed. Many studies do not give sufficient details about the exact procedures they use, insufficient information about how the target is conceptualised and very little information about the data they collect or the statistics they run, if any. Most studies lack sufficient information to attempt proper scientific replicability, which hinders the possible advantage of having those unsuccessful studies published and fuels doubt and apprehension in the scientific community. The major weakness remains the lack of transparency and detailed procedures. Regarding the data and the studies, the results of the *ad-hoc* quality assessment of the group studies revealed that the overall evidence found was of weak-to-moderate quality and any generalisation warrants precaution. The clinical interest, however, remains at the centre of this review, and we sketch some prudent recommendations based on the overall findings in the next section.

### Clinical Recommendations

It is critical for the practitioner and the researcher to properly select the suitable candidates for EMDR therapy and conceptualise their cases (e.g., choice of targets). They should question at all times, which are the adequate protocol adaptations, what should be the most favourable frequency of application, the reasonable number of sessions and their length.

There was overall a high number of studies reporting positive treatment effects in Pain, Anxiety, Mood (e.g., depression), and Stress-related disorders suggesting that EMDR is an appropriate treatment, at least as a complementary option. In cases where standard care is ineffective, EMDR seems to be a plausible alternative. Moreover, EMDR could be of interest in populations for which classical psychotherapy using speech and oral communication is limited or difficult (e.g., Dementia, Aphasia). For instance, EMDR may be suitable to use when there is a language barrier between the practitioner and the patient, also in young infants, deafness and speech disabilities. Furthermore, EMDR can help treat when treatment as usual (e.g., exposition therapy, radiotherapy) cannot be followed due to psychological reasons. Finally, due to the reported rapid action, it can also be used as first-line treatment in migraine or acute pain situations.

Unfortunately, we did not find studies on tobacco addiction, although very brief protocols inspired by EMDR seem to be promising in treating relapse after tobacco cessation (Tsoutsa et al., 2013). We believe that more studies on affect and affective disorders, such as narcissistic personality disorder (e.g., Mosquera and Knipe, 2015) and antisocial personality disorder, should also be encouraged.

### Future Directions

Despite the general limitations, EMDR -as most psychotherapeutic approaches- remains a highly personalised, tailored therapeutic tool, and it should be understood as such. Future research, however, should try to understand what is operational in EMDR, mainly because of the myriad of protocols, differences found between studies, the heterogeneity in session length, their number and the conceptualisation of the targets.

Moreover, future research could try better ways to compare the therapy to other alternatives and active treatments (vs Waiting lists). A possibility is the use of a multi-methods approach where qualitative and quantitative methods are used across time, with the help of structural and functional imaging techniques. Special care should be taken in future RCTs and controlled group studies by selecting appropriate sample sizes and systematically reporting follow-ups and dropout rates.

### Limitations of the Review

The major limitation of this review remains the difficulty of selecting studies where trauma was not measured, was not the aim of the therapy or were patients not included based on trauma diagnosis. Despite our careful selection of studies not focusing on trauma, in some cases, the authors concluded that the symptoms treated were very likely related to traumatic experiences.

Another significant limitation is that by attempting to be highly inclusive on works on EMDR, some potentially relevant studies were undoubtedly left out (e.g., studies using BLS only but not the entire EMDR protocol).

## Concluding Remarks

The present review found that EMDR can successfully treat several disorders beyond PTSD and Trauma. Results shed light on several aspects that support the interest of its practise in mental health care. Despite the clear need for more rigorous research, our review also demonstrated that EMDR has translational interests. The fact that this therapy could be helpful in non-pathological situations (e.g., performance) broadens the scope of its benefits and invites for interdisciplinary research. Also, because of its potential advantages, we believe that EMDR could be considered in major crisis situations, such as to alleviate the imminent and disproportionate mental health sequelae of a world pandemic (Ridley et al., 2020) that has left grief, despair, frustration and affective pain.

## Data Availability Statement

The datasets presented in this study can be found in online repositories. The names of the repository/repositories and accession number(s) can be found in the article/Supplementary Materials.

## Author Contributions

CS conceptualised the study. LB designed the study. Literature search, analyses, and manuscript writing was done by CS and LB. All authors contributed to the article and approved the submitted version.

## Conflict of Interest

The authors declare that the research was conducted in the absence of any commercial or financial relationships that could be construed as a potential conflict of interest.

## Publisher's Note

All claims expressed in this article are solely those of the authors and do not necessarily represent those of their affiliated organizations, or those of the publisher, the editors and the reviewers. Any product that may be evaluated in this article, or claim that may be made by its manufacturer, is not guaranteed or endorsed by the publisher.
