# Supplementary material for: EMDR as Treatment Option for Conditions Other Than PTSD: A Systematic Review
Source: Front Psychol. 2021 Sep 20;12:644369. doi: 10.3389/fpsyg.2021.644369 (PMC8488430; doi:10.3389/fpsyg.2021.644369)
Supplement: Supplementary file 1 [file Table_1.docx]

**Supplementary material**

*“EMDR as a treatment option for conditions other than PTSD: A systematic review”*

Charles Scelles and Luis C. Bulnes.

**Table.S1. Characteristics of studies related to Mood Disorders**.

**Abbreviations Table 3:** MDD= Major Depressive Disorder. TAU= Treatment as Usual. BDI=Beck Depression Inventory. BAI=Beck Anxiety Inventory. TAU=Treatment as Usual. SCL-90R= Symptom Checklist 90 Revised. GSI= Global Severity Index. TSC-40= Trauma Symptom Checklist 40. QLI= Quality of Life Index. TF-CBT=Trauma Focused Cognitive Behavioural Therapy. MADRS= Montgomery–Åsberg Depression Rating Scale. PSQI=Pittsburgh Sleep Quality Index. MINI-ICF-APP=Mini International Classification of Functioning Scale. WHOQOL-BREF: World Health Organisation Quality of Life-Short Version. BSSI= Beck Scale for Suicidal Ideation. MI=Myocardial infarction. PHQ-9= Patient health questionnaire.

|  | **Topic** | **Design** | **Population** | **Treatment and Setting** | **Number of Sessions** | **Measures** | **EMDR protocol** | **Sample Size, n** | **Main Result** | **Secondary Outcome** | **Other /adverse effects** |
| --- | --- | --- | --- | --- | --- | --- | --- | --- | --- | --- | --- |
| **Hofmann et al (2014)** | Depression | Non-randomized controlled exploratory study | Patients with unipolar primary depression | EMDR vs CBT  Targets: stressful events linked with the depression Follow-up: unspecified | 3 to 16 sessions | Depression (BDI) | Standard | 42  (21 EMDR; 21CBT) | Reduced depression scores in both groups  (ps’<0·001).  Greater improvement in EMDR vs CBT (p=0·011). | Higher remissions in EMDR group (p<0·001). | ·· |
| **Hase et al(2015)** | Depression | Group controlled | Patients with depressive episodes | EMDR+ TAU vs control. therapy and treatment as usual (TAU).  Targets: disturbing memories related to the onset and course of depression.  Follow-up: 12 and 16 months | Average of 4.6 sessions as 1 to 2 x60 min. EMDR weekly. | Depressive symptoms (SCL-90-R) Depression severity (GSI) | Standard. | 48  (32 EMDR; 16 Control) | Greater reduction in depressive symptoms in EMDR group (SCL-90R) (p=0·015, d=1·18)  Results maintained at 1 year follow up, with less relapses. | .. | .. |
| **Gauhar (2016)** | Depression | RCT | Patients with major depressive disorder  (MDD) | EMDR vs waiting list.  Targets: disturbing events related to depression. Follow-up: 3 months | 6-8 sessions | Depression (BDI), Trauma and distress (TSC-40), Quality of life (QLI) | Standard | 17  (10, EMDR; 7, Waiting list) | Reduced depressive scores, greater in EMDR group (p= ·001, d = 1·97). | Greater improvement in distress scores change for EMDR (p<0·001, d =1·57). and quality of life (p<0·001; d =2·16) | Rapid effectiveness of EMDR. |
| **Ostacoli et al (2018)** | Depression | RCT | Patients with recurrent depressive episodes | EMDR vs CBT as adjunctive treatments of antidepressant medication (ADM)  Targets: factors of emergence, maintenance, and recurrence of depressive episodes, triggers of the current depressive episode, beliefs, depressive and suicidal states.  Follow-up: 6 months | 15 ± 3 individual sessions of EMDR or CBT,  Follow- up: 6-months. | Depression (DBI) | DeprEnd protocol | 66 (31, EMDR, 35, CBT) | Reduced depressive scores in both groups (p < 0·001).  No difference in outcome between groups.  Results maintained at 6mo follow up. |  | Faster improvement in EMDR group |
| **Minelli et al (2019)** | Depression | RCT | Patients with Treatment Resistant Depression  (TRD) and MDD. | EMDR vs TF-CBT.  Targets: unspecified Follow-up: 24 weeks | 3x60min. sessions (EMDR or TF-CBT) per week for 8 weeks. | Depression (MADRS; BDI) Anxiety (BAI), Sleep (PSQI), Social functioning scale (MINI-ICF-APP). | Standard | 22 (12, EMDR; 10, CBT) | Reduced depression in EMDR and TF-CBT (p<0·001).  Improvement in all measures for both treatments (all ps’<0·001).  Results maintained at follow-up in EMD group only.  (MADRS (p= 0·04) BDI (p= 0·02). |  | Many MDD and TRD patients have a PTSD comorbidity |
| **Jahanfar et al (2020)** | Depression | RCT | Patients with MDD and history of trauma. | EMDR vs No intervention (control)  Targets: unspecified Follow-up: no follow-up | 8x 90 minutes sessions over 3 weeks. Control: no intervention | Quality of Life (WHOQOL-BREF) | Standard | 70  (35, EMDR; 35, control). | Improvement of quality of life in both groups (all ps’<0·01).  Greater improvement in EMDR (all ps’ <0·002). | .. | ·· |
| **Rosas Uribe et al(2010)** | Depression  Specific aspects | Case Study | Patients with unipolar major depressive disorder | Targets: negative experiential events that contributed to their emotional disorder.  Follow-up: no follow-up | 12 sessions EMDR and emotional identification tasks. | Depression (BDI) | Standard | 3 | Reduced depression symptoms (BDI) in all patients. | Improved emotional cognitive processing and on long-term memory conceptual organization. | **BDI scores at baseline showed patients had mild to moderate depression.* |
| **Fereidouni et al (2019)** | Depression  Specific aspects | RCT | Patients with MDD who exhibited suicidal thoughts. | EMDR vs TAU  Targets: memories, beliefs, sensations, images, symbols related to suicidal thoughts.  Follow-up: no follow up | 9 sessions of 45–90 min, 3 days per week for 3 weeks | Suicide Ideation (BSSI) | Standard | 70 (35, EMDR; 35, TAU) | Reduced suicide ideations in EMDR only (p<0·001). | Suicidal thoughts were often related to childhood traumatic memories | ·· |
| **Behnammoghadam et al (2015)** | Depression  Subtypes | RCT | Patients with depression and history of MI. | EMDR vs No-Treatment (control).  Targets: most terrible memories of cardiac incident; related unreasonable beliefs.  Follow-up: unspecified | 3 weekly sessions of 45–90 minutes, during four months after the MI. | Depressive symptoms (BDI) | Standard | 60 (30 EMDR; 30, control) | Reduced depressive symptoms in EMDR group (p<0·001).  Increase of depressive symptoms in control (p<0·001). | ·· | .. |
| **Guina and Guina (2018)** | Depression  Subtypes | Case Report | Patient with post-stroke depression and expressive aphasia. | Targets**:** memories related to stroke, a suicide attempt and an unidentified trigger “25 years ago”.  Follow-up: unspecified | Weekly 1-hour sessions over two months 24 months after stroke. | Depression severity (PHQ-9) | Standard | 1 | Reduced depression severity (PHQ) in 4 sessions. (scores from 24 to 5). | Improvement in aphasia. | ·· |
| **Carletto et al (2017)** | Depression  Subtypes | Review | .. | Efficacy of EMDR in depression | .. | .. | .. | 7 | EMDR may be beneficial for depression. | Overall proper research methodology is lacking |  |

**Table.S2. Characteristics of studies related to Reaction to Severe Stress**.

**Abbreviations Table 4:** HADS= Hospital Anxiety and Depression scale. SOC-29=Sense of coherence.BT=Before Treatment. VAS=visual analogue scale. BDI=Becks’ depression and anxiety scale. PRAQ=Pregnancy Related Anxiety Questionnaire.

|  | **Topic** | **Design** | **Population** | **Treatment and Setting** | **Number**  **of Sessions** | **Measures** | **EMDR protocol** | **Sample Size, n** | **Main Result** | **Secondary Outcome** | **Other /adverse effects** |
| --- | --- | --- | --- | --- | --- | --- | --- | --- | --- | --- | --- |
| **Trznadel (2017)** | Cancer | Case  Study | Patient with malignant neoplasm of the breast with pre-existing feelings of helplessness and anxiety. | Targets: Adverse Life Event (taking a university entrance examination when she was 19 years old) Follow-up: 2, 4, 6 and 17 months | 3 weekly 90-min sessions | Self-Report (Distress Thermometer, Checklist of problems) | Standard | 1 | Reduced levels of distress. | ·· | ·· |
| **Szpringer (2018)** | Cancer | Non-RCT | Female patients with Glioblastoma Multiforme, diagnosed since 2 to 3 months | EMDR vs Control  Effects of EMDR on anxiety, depression and sense of coherence.  Targets: unspecified.  Follow-up: 4 and 8 months | 10–12 individual sessions for 4 months | Depression (HADS), Overall life- stress cognitions (SOC-29), Patient Caregivers Questionnaire. | Standard | 37  (18, EMDR; 19, Control) | Reduced anxiety (d=2·11), depression (d=2·25), and anger symptoms (d=0·97) (respectively 0·47, 0·63 and 0·20 in control group).  Confirmed by caregivers’ assessment. | Increased sense of coherence in EMDR group (p<0·001).  Decreased sense of coherence in control group (p<0·024) | ·· |
| **Dinapoli (2019)** | Cancer | Case Report | Male patient with Carcinoma of larynx reporting severe anxiety related to the use of thermoplastic mask necessary for radiotherapy (RT) | Targets: memories of a locked room impossible to open with a key. Follow-up: no follow-up | 3 sessions of 60 minutes in 2 weeks.  + venlafaxine 75 mg/day and lorazepam 1 mg/day. | Self-Report (Distress Thermometer, Visual Analog Scale -VAS-), HADS, | EMDR protocol for oncology | 1 | Reduced mask anxiety after third session and successfully proceeded with oncological evaluation. Radiotherapy started and was performed for 35 consecutive days with a good tolerance, and without interruptions. | Distress was 3/10 (9/10 BT); anxiety was 8/21 (20/21 BT); and depression was 6/21 (12/21 BT). Intolerability VAS was 1 (10 BT). | .. |
| **Borji (2019)** | Cancer | RCT | Patients with gastrointestinal cancer | EMDR at home by a nurse vs routine care at home by families (Control).  Targets: memories linked to the cancer.  Follow-up: no follow-up | Home Care including 2 EMDR sessions | Perceived Stress Scale | Standard | 60  (30, EMDR; 30, Control) | Reduced distress after intervention (P<0·05). | ·· | .. |
| **Hatefi (2019)** | Other (Injuries) | RCT | Patients suffering from Spinal Cord Injuries. | EMDR vs Control  Targets: a scene or a memory of the disease that caused the mental disturbance  Follow-up: no follow-up | 2 sessions, 45-60min each. | Depression and anxiety (BDI) | Standard | 68  (33, EMDR; 35, Control) | Reduced  depression scores, from 45·36 to 40·54 (p < 0·02).  and anxiety scores  from 43·15 to 20·03  (p < 0·001).  Reduced depression (p<0·04) and anxiety(p<0·001) in EMDR group. | Higher reduction of anxiety vs depression  (p < 0·001). |  |
| **Rahimi (2019)** | Other (Haemodialysis) | RCT | Patients receiving haemodialysis three times a week at least for 6 months, without stressful life events in the previous 6 months | EMDR intervention during haemodialysis vs routine care (Control).  Targets: not reported Follow-up: no follow-up | 3 weekly sessions x 2 weeks | Anxiety and depression (HADS) | EMDR Phase 4 only | 90  (45, EMDR; 45, Control) | Reduced anxiety (p<0·05) and depression (p<0·05) after treatment. | ·· | .. |
| **Zolghadr (2019)** | Other (Childbirth Anxiety) | RCT | Multiparous women in the next normal pregnancy, following a prior stillbirth. | EMDR at admission for delivery vs routine care (control)  Targets: previous experience of stillbirth  Follow-up: no follow-up | 1x 90min session | Anxiety (PRAQ) | ·· | 30  (15, EDMR; 15, Control) | Reduced anxiety compared to control group (p < 0·05) and across time (p < 0·01). | .. | .. |
| **Shapiro (2014)** | Other | Review | .. | Effects of EMDR in adverse life experiences. | .. | .. | .. | 24 studies | Twenty-four RCTs support the positive effects of EMDR therapy in the treatment of emotional trauma and other adverse life experiences and Somatic Manifestations |  | .. |

**Table. S3. Characteristics of studies related to Anxiety Disorders**.

**Abbreviations Table 5:** SPQ= Spider Phobia Questionnaire. BAT=Behavioural avoidance Test. MINI =Mini International Neuropsychiatric Interview. SCL-90R= Symptoms Check list-90-revised. BDI=Beck depression inventory. BAI=Beck anxiety inventory. ACQ=Agoraphobic cognitions questionnaire. BSQ=Body Sensations questionnaire. MI=Mobility inventory for agoraphobia. BSI=Brief Symptom inventory.DS=Distress scale. BBSIQ= Body sensations interpretation questionnaire. PAI= Panic appraisal inventory. SAS=social adjustment scale. PDSS= Panic disorder symptom severity interview. STAI-Y=State Trait Anxiety Inventory. PAAAS= Panic Attack and anticipatory anxiety. MSPS= Marks-Sheehan Phobia Scale. DISS= Disability Social Scale. SUD= subjective units of disturbance. VOC= Validity of positive cognitions. GADQ-IV= The Generalized Anxiety Disorder Questionnaire. PSWQ= Pennsylvania State Worry Questionnaire. IUS= Intolerance of uncertainty questionnaire. CAQ= Cognitive Avoidance Questionnaire. LSAS= Liebowitz social anxiety scale.

|  | **Topic** | **Design** | **Population** | **Treatment and Setting** | **Number of Sessions** | **Measures** | **EMDR protocol** | **Sample Size, n** | **Main Result** | **Secondary Outcome** | **Other /adverse effects** |
| --- | --- | --- | --- | --- | --- | --- | --- | --- | --- | --- | --- |
| **Muris and Merckelbach (1995)** | Phobia | Case Study | Two female patients with arachnophobia | Targets: Chosen by therapist. Memories related to aversive confrontation with phobic object. Follow-up: no follow-up | 3 x 1-hour session followed by 1 x 2.5-hour in vivo exposure | Spider reported Phobia (SPQ)  Spider avoidance (BAT) | EMDR protocol for phobia | 2 | Reduced fear of spiders (SPQ)  Possible to touch spider after EMDR for both patients (BAT). | .. | .. |
| **de Jongh (2012)** | Phobia | Case Study | Female patient with emetophobia | Targets: Childhood memory of vomiting in public. Follow-up: 3 year | 4 sessions EMDR | Emetophobia and comorbid symptoms (MINI) and Phobia symptom severity (SCL-90-R) | EMDR protocol for phobia-  Auditory alternations. | 1 | Reduced symptoms severity to non-clinical (SCL-90) scores from 275 to 121. No relapse after 3-year follow up. | Negative cognitions about vomit remained, with no panic to vomit. | .. |
| **Lapsekili and Yelboga (2014)** | Phobia | Case Study | Female patient with aviophobia | Targets: memories related to in-flight turbulence and fear of dying. Follow-up: 1 and 6 months | 3 x 1-hour sessions | Ability to take a plane | EMDR protocol for phobia | 1 | Reduced distress of flying and ability to take a plane after third session. No relapse at one- and six-months follow-up. | ·· | .. |
| **Goldstein and Feske (1994)** | Panic Disorder | Case Series | Outpatients with panic disorders and agoraphobia. | Targets: anxiogenic memories related to worst panic attack, and anticipated panic episodes.  Follow-up: unspecified | 5 x 90 minutes sessions over 2 weeks | Depression (BDI), Anxiety (BAI) Agoraphobia (ACQ), Body Sensations (BSQ), Mobility (MI), General Distress (BSI), Distress in daily functioning (DS), Severity of symptoms (SCL-90) and behavioural self-monitoring records. | Standard | 7 | Improvement in all measures (all p<0·028). Reduced frequency of panic attacks. | ·· | .. |
| **Goldstein et al (2000)** | Panic Disorder | RCT | Outpatients with Agoraphobia and Anxiety for over 1-year. | EMDR vs waiting list vs placebo (same amount of therapist contact as EMDR).  Targets: Anxiogenic memory related to worst or first panic attack, or a fearful body sensation. Follow-up: 1 month. | 6x 90 minutes over an average of 4 weeks | Agoraphobia (ACQ) Body Sensations (BSQ), Body Interpretation (BBSIQ), Panic Appraisal (PAI), Mobility (MI), Depression (BDI), Anxiety (BAI), General distress (BSI), Social functioning (SAS) Distress in daily life (DS), Panic Symptom Severity (PDSS), + Self-Monitoring | Standard | 46  (18, EMDR;13, Placebo; 14 wait) | Reduced panic (PDSS) agoraphobia (ACQ) symptoms severity and self-monitoring vs waiting list (ps < 0·05). Not for number of panic attacks (p > 0·10) | Differences between EMDR and placebo were not significant on any measure (η2 = 0·00-0·06). | ·· |
| **Fernandez and Faretta (2007)** | Panic Disorder | Case Study | Female patient with panic disorder and agoraphobia for over 10 years | Targets: Patient's first panic attack Follow-up: 1 year | 15 sessions EMDR, including three preparation sessions, and three on enhancement of future behaviours | Self-Report in a diary, with monitoring of panic attacks, and behaviours, cognitive, physiological, emotional factors. | Standard | 1 | Reduced and resolved symptoms after 15 sessions. Results were maintained at 1-year follow-up. | Improved addressing future behaviours and resolving anticipation anxiety. Gradually resumed functions. | ·· |
| **Faretta (2012)** | Panic Disorder | Group controlled | Patients with Panic disorder with agoraphobia | EMDR vs CBT.  Targets: Various linked to the panic disorder Follow-up: 3 and 12 months | 12 sessions | Anxiety (STAI-Y), Panic and Anticipatory Anxiety (PAAAS), Phobia (MSPS), Social function (DISS), Severity of symptoms (SCL-90-R).  Only descriptive analysis. | Standard | 20  (10 EMDR; 10, CBT) | Improvement in all measures after 12 sessions.  Symptoms progression similar in both groups. | EMDR shows a qualitative faster progression in symptom reduction. Results maintained at follow-up. | ·· |
| **Bhagwagar (2016)** | Panic Disorder | Case Study | Patient diagnosed with Panic Disorder with Agoraphobia | Targets: diverse, Memories linked to panic attacks, childhood and future templates. Follow-up: 6 months - 1 year - 5 years | 17 sessions, including 6 preparation sessions | EMDR self-reported scales (SUD, VOC) | Leeds Model | 1 | Absence of panic attacks after 17 session, and reduction of agoraphobic symptoms.  Results maintained at 1- and 5-years follow-up | Improvement in well-being: calmness, cheerfulness, and confidence both at work and family. |  |
| **Nicolas and Vautier (2017)** | Panic Disorder | Case Study | Patient suffering from panic disorder with agoraphobia for more than 20 years | Targets: Thought trigger causes and anxiety of difficulty to orient during a fire.  Follow-up: 1 and 8 months | 1 session EMDR | Diagnostic Criteria for DSM-IV, Behaviour and self-report | Standard | 1 | Resolved reported avoidance, Absence of panic attacks. 1mo after treatment, patient no longer met diagnostic criteria for Panic Disorder with Agoraphobia. Results maintained at 8 months follow-up. | EMDR therapy allowed emergence of previously non identified traumatic material, which could then be treated. | ·· |
| **Farima et al (2015)** | Generalized Anxiety Disorder | Group Study | Patient with General Anxiety Disorder | Targets: unspecified Follow-up: 1 month | Multiple 90 min sessions | Anxiety (GADQ-IV), Distress (PSWQ), the Worry Domain Questionnaire (WDQ), Uncertainty (IUS), Avoidance (CAQ) | Standard | 3 | Reduction of all measure’s vs first session and baseline. Results maintained at 1-month follow-up. | ·· | ·· |
| **Sagaltici and Demirci (2019)** | Social Anxiety Disorder | Case Report | Patient with social anxiety disorder. | Targets: Adverse life event (Embarrassing memory of getting wet in public and getting beaten up at 6 by peer) Follow-up: 6 months. | 4 EMDR sessions | Social anxiety (LSAS), Depression (BDS) and anxiety (BAS) | Standard | 1 | Reduced social anxiety (LSAS) from 144 to 76, Reduced depression (BDS) scores from 48 to (..) and reduced anxiety (BAS) scores from 35 to 17 after 4 sessions. 6mo follow up LSAS scores were 58, BDS were 9 and BAS were 13. | ·· | ·· |
| **Faretta and Leeds (2017)** | Panic Disorder | Review |  | Effects of EMDR in panic disorders. | ·· | ·· | ·· | 5 studies | One pilot study and series of individual case reports suggest that EMDR is effective in eliminating symptoms of panic and agoraphobia, whereas two studies with placebo controls failed to show comparable outcomes. | .. | ·· |
| **Faretta and Farra (2019)** | Anxiety Disorders | Review |  | Effects of EMDR in anxiety disorders | ·· | ·· | ·· | 6 studies | Data suggest EMDR therapy may be effective not only for PD but also for specific phobias. | .. | ·· |
| **Yunitri et al (2020)** | Anxiety Disorders | Meta  Analysis | .. | A manual and systematic search using various databases and reference lists of systematic review articles published up to December 2018 was conducted. | ·· | ·· | ·· | 17 studies | EMDR is efficacious for reducing symptoms of anxiety (g = -0·71; 95% CI: -0·96 to -0·47), panic(g = -0·62; 95% CI: -1·10 to -0·14), phobia(g = -0·45; 95% CI: -0·81 to -0·08),, and behavioural/somatic symptoms (g = -0·40; 95% CI: -0·63 to -0·12), but not traumatic feelings (g = -0·48; 95% CI: -1·14 to -0·18). | ·· | ·· |

**Table.S4. Characteristics of studies related to Performance Anxiety**.

**Abbreviations Table 6.** ADIS-R= Anxiety Disorders Interview Schedule. PRCA-24= Personal report of communications anxiety. PRPSA=Personal report of public speaking anxiety. BASA= (observer)-Behavioural assessment speech anxiety. PRCS= personal report of confidence as a speaker. SUD= subjective units of discomfort. SCL

Skin conductance level. SR=stimulus response inventory. TAI= Test anxiety inventory. STAI=State Trait Anxiety inventory. FNE= Fear of negative evaluation scale. BDORT=Bi-digital-O-ring-Test. LS=Likert scale. CSAI-2R= Competitive state anxiety inventory-2R. IFES=Impact of future events scale. SVQ= Social validation questionnaire. CH= cognitive hypnotherapy. ABRSM= Associated Board of the Royal Schools of Music. SRQ= Self report anxiety. PANAS=Positive and negative affect scale.

|  | **Topic** | **Design** | **Population** | **Treatment and Setting** | **Number of Sessions** | **Measures** | **EMDR protocol** | **Sample Size, n** | **Main Result** | **Secondary Outcome** | **Other /adverse effects** |
| --- | --- | --- | --- | --- | --- | --- | --- | --- | --- | --- | --- |
| **Foley**  **and Spates (1995)** | Public Speaking Anxiety | RCT | Students suffering from public speaking anxiety | EMDR (eye mvmts) vs EMDR (sound stimulus) vs EMDR (Eye resting fixation) and control (no treatment).  Targets:  specific distressing speech-related event.  **Follow-up:** 1 week | One or Two sessions. | Anxiety (ADIS-R) Communication Anxiety (PRCA-24), Public Speaking Anxiety (PRPSA), Observed speech Anxiety (BASA), Heart Rate Reactivity. | Standard | 40  (10 x 4 groups) | Improvement of communication anxiety (PRCA-24) in all treatments (p<0·0001). except the no treatment control.  All EMD interventions improved vs control except the sound condition. | The eye component was not essential to overall improvement. | ·· |
| **Carrigan and Levis (1999)** | Public Speaking Anxiety | RCT | Undergraduate psychology students | EMDR1 (fear imagery and eye mvmts) vs. EMDR2 (relaxing imagery and eye mvmts) vs Control1 (fear imagery) vs Control2 (relaxing imagery).  Targets: Representation of the entire fear of public speaking  Follow-up: no follow-up | One session | Anxiety (Subjective discomfort of imagined Targets(SUD)). Confidence as a Speaker; (PRCS). Physiological anxiety (SCL). | Standard | 71  (18, EMDR1; 18, EMDR2; 18, Control1; 17, Control2). | No significant treatment differences.  Reduced SUDs of discomfort of fearful imagery, and Reduced physiological anxiety (SCL) but  No reduction of public speaking anxiety. | ·· | ·· |
| **Aslani et al (2014)** | Public Speaking Anxiety | Group controlled | Students with speech anxiety | EMDR vs No treatment (control).  Targets: imagined traumatic image, unrelated to public speaking.  Follow-up: no follow-up | 7 sessions of 90min | Confidence as a speaker (PRCS), Anxiety (SR) | Standard | 30  (15, EMDR; 15, Control) | Improved confidence of public speaking  (p=0·001) reduced reported speech anxiety (p=0·001) | ·· | ·· |
| **Maxfield and Melnyk (2000)** | Test Anxiety | Group controlled | Students with test-anxiety | EMDR vs Wait List.    Targets: memory of first, worst, most recent, and future (anticipated) experiences of test anxiety Follow-up: 2 months | Single 90 min session | Test Anxiety (TAI), Anxiety (STAI), Evaluation Fear (FNE) | Standard | 17  (8, EMDR; 9, Wait). | Reduced anxiety at post-treatment (d=1·08), and reduced evaluation fear vs. waiting list (p=0·05) (FNE). Evaluation fear results were maintained at follow-up (p<0·05). | .. | ·· |
| **Rathschlag and Memmert (2014)** | Performance Anxiety | RCT, pilot | Normal participants | EMDR vs. No intervention (control)  Targets: autobiographical memory of anxiety. Follow-up: 2 weeks | Single 60-120 min session | Anxiety (STAI). subjective  Anxiety (LS), Physical performance (BDORT) | Advanced EMDR Protocol (wingwave) | 50  (25, EMDR; 25, Control) | Reduced subjective anxiety (LS, p<0·008) and state (p<0·022). and trait (p=0·035) anxiety (STAI).  Increased physical performance (p<0·001). | .. | ·· |
| **Falls et al (2018)** | Performance Anxiety | Case series | Competitive golfers, with performance anxiety (prospective performance). | Targets: troubling prospective imagery related to their golf Follow-up: different follow-up between participants, between 1 and 8 weeks | 3 weekly sessions | Competition Anxiety (CSAI-2R), Prospective negative imagery (IFES). Social validation and significance of intervention (SVQ) | Standard | 4 | Reduced anxiety overall (CSAI)  Reduced impact of prospective memory. Overall acceptance and significance of intervention. | .. | ·· |
| **Brooker (2018)** | Performance Anxiety | Group controlled | Advanced pianists with music performance anxiety | EMDR vs CH vs control group.    Targets: “feared situation”. Follow-up: no follow-up | 2 weekly sessions, in between concerts. | Performance assessment (ABRSM), Anxiety (STAI), Subjective anxiety (SRQ) | Standard | 46  (Randomly assigned, final samples not reported) | Reduced anxiety (STAI) in both EMDR and CH vs control (p<0·005).  Reduced subjective anxiety (SRQ) in EMDR vs control (p=0·007).  Improvement of performance in EMDR (p=0·012), and CH(p=0·022) vs control. | .. | ·· |
| **Vauthier et al (2019)** | Test Anxiety  /Learning | RCT | Students with disturbing and disagreeable memories related to learning mathematics. | EMDR vs control (information about relaxation techniques that could be used before a math test).  Targets: anxiety-inducing memory linked to the learning of mathematics Follow-up: 1 month | Single 90 min session | Affective bias and congruency Positive and (PANAS), Subjective performance efficacy (LS). | Standard | 40  (20, EMDR; 20, Control) | Reduced negative emotions of mathematics (p<0·001) and an increase in the strength of their self-efficacy (p<0·0001).  Results maintained at follow-up. | ·· | ·· |

**Table.S5. Characteristics of studies related to Obsessive-Compulsive Disorder**.

**Abbreviations Table 7:** SSRI=Selective serotonin reuptake inhibitor. YBOCS= Yale-Brown obsessive-compulsive scale. BAI= Becks anxiety inventory. BDI= Becks depression inventory. PI= Padua Inventory. OBQ=Obsessive beliefs questionnaire. DES= dissociative experience scale. OCI=obsessive compulsive inventory. PHQ-9=Patient Health questionnaire-depression module. GAD-7= general anxiety disorder. WSAS= Work and Social Adjustment Scale.

|  | **Topic** | **Design** | **Population** | **Treatment and Setting** | **Number of Sessions** | **Measures** | **EMDR protocol** | **Sample Size** | **Main Result** | **Secondary Outcome** | **Other /adverse effects** |
| --- | --- | --- | --- | --- | --- | --- | --- | --- | --- | --- | --- |
| **Corrigan and Jennett (2004)** | OCD | Case study | Patient with history of OCD that did not respond to CBT, Fluoxetine, Paroxetine, Clomipramine and Amitriptyline. | Observational study of effects of EMDR and ephedra alkaloids. Mindfulness,  Targets: memories of childhood sexual abuse  Follow-up: 9 months | 7 sessions | ·· | ·· | 1 | Reduction of OCD symptoms and reduction of medication (Fluvoxamine 300mg to 50mg). | Patient relapsed 9 months later after taking an alkaloid. |  |
| **Bohm and Voderholzer (2010)** | OCD | Case study | Patients with OCD | EMDR and Confrontation therapy.  Follow-up: 6 months | Various number of sessions | ·· | Standard | 3 | Reduced OCD symptoms in all patients by about 60%. | Improved motivation, and emotional management. |  |
| **Nazari et al (2011)** | OCD | RCT | Patients with OCD | EMDR vs Citalopram (SSRI) for 12 weeks.  Targets: unspecified Follow-up: unspecified | 12 weeks of treatment | Obsessive thoughts and behaviours (YBOCS) | Standard | 60  (30, EMDR; 30, SSRI) | Reduced OCD symptoms in both groups (ps’<0·001). Greater reduction in EMDR vs Citalopram (p<0·001). | ·· |  |
| **Marr (2012)** | OCD | Case reports | Patients with OCD | Targets: current OCD triggers, future templates, past-related memories Follow-up: 4-6 months | 14 to 16 sessions | Obsessive thoughts and behaviours (YBOCS) | EMDR Phobia Protocol | 4 | Reduced OCD symptoms,  Reduced reported distress -results maintained at 4–6 months follow-up. | Improvements were reported as from first two to three sessions. |  |
| **Marsden (2016)** | OCD | Case report | Patients with OCD | Targets: current OCD trigger, future templates, past-related memories Follow-up: 6 months | 16 sessions | Obsessive thoughts and behaviours (YBOCS) | EMDR Phobia Protocol | 3 | Reduced OCD symptoms (YBOCS) in 2/3 patients- Only slight improvement in patient3.  Results maintained at Six-month follow-up | Overall improvement of subjective confidence and less shame to talk about their experiences. |  |
| **Mazzoni et al (2017)** | OCD | Case report | Patients with OCD | Combined EMDR and Exposure therapy  Targets: Traumatic memories Follow-up: 3- and 6-month follow-up | 20 to 45 sessions. | Obsessive thoughts and behaviours (YBOCS), Anxiety (BAI), Depression (BDI), Obsessions and compulsions (PI), obsessive beliefs (OBQ). dissociative symptoms (DES) | EMDR protocol for OCD (Pozza et al. 2014) | 3 | Reduction of OCD symptoms in all three patients in all variables. | EMDR was able to facilitate use of exposure therapy and homework. |  |
| **Marsden et al (2018)** | OCD | RCT | Patients with OCD | EMDR vs CBT  Targets: current triggers (obsessions and compulsions separate from recent traumatic events); future templates (prospective successful action); and past disturbing events.  Follow-up: 6 months | 16 sessions | Obsessive thoughts and behaviours (YBOCS; OCI), Depression (PHQ-9), generalised Anxiety (GAD-7), Functioning (WSAS) | EMDR Phobia Protocol | 55  (29, EMDR; 26, CBT) | No clinically significant differences between treatments at post-treatment (p>0·05). Overall, only 30% of patients improved in OCD symptoms. |  |  |

**Table.S6. Characteristics of studies related to Pain**.

**Abbreviations Table 8.** PLP=Phantom limb pain. MPQ=McGill Pain Questionnaire. BDI=Beck depression inventory. SFMPQ: short form McGill pain Questionnaire. CSQ=coping skills Questionnaire. SF-36= Short form health survey. STAI= State Trait anxiety questionnaire. BDI= Beck depression inventory. SCID-II= Structure clinical Interview for DSM. VAS= Visual analogue scale. PCL-S= Post-traumatic checklist scale. PBPI= Pain beliefs and perceptions inventory. RAPS= Rheumatoid arthritis pain scale. MISS=methotrexate intolerance severity score. IES= Impact of Event Scale. SCL-90= Symptom Checklist 90.CIS-20= Checklist Individual Strength-Revised. SIL= Self-Inventory List. CES= Center for Epidemiologic Studies Depression Scale. FIQ=Fibromyalgia impact questionnaire. FS=fatigue scale. BDI=Beck anxiety inventory. FMS= fibromyalgia syndrome. PSQI=Pittsburg Sleep Quality index. STAS= State-Trait Anger Scale. PDS= The Posttraumatic Diagnostic Scale. SPL=subjective pain level. MIDA= Migraine Dis-ability Assessment Scale.LS= Likert scale. MAACL= Multiple Affect Adjective Check List.

|  | **Topic** | **Design** | **Population** | **Treatment and Setting** | **Number of Sessions** | **Measures** | **EMDR protocol** | **Sample Size, n** | **Main Result** | **Secondary Outcome** | **Other /adverse effects** |
| --- | --- | --- | --- | --- | --- | --- | --- | --- | --- | --- | --- |
| **Ray and Page (2002)** | Chronic Pain | RCT | Patients with chronic pain | Hypnosis followed by EMDR or EMDR followed by hypnosis  Targets: pain, negative cognitions related to pain, noxious images or feelings associated with the pain  Follow-up: unspecified. | Single Session each, 1x week. | Pain perception (MPQ), depression (BDI) | Standard | 15 | Reduced overall pain perception scores, but not significant (p=0·06). | Patients preferred hypnosis (87%). | .. |
| **Grant and Threlfo (2002)** | Chronic Pain | Case study | Patients with chronic pain | Targets: memory of the first time they experienced the pain and associated negative thoughts and feelings  Follow-up: 2 months | 9 weekly sessions | Pain perception (SFMPQ); Negative construals-coping (CSQ); Self reports, at Baseline, at treatment and then after 2months. | Pain protocol + standard | 3 | Reduced pain level.  Improvement in negative cognitions (CSQ)  Results maintained after 2 months. | ·· | ·· |
| **Mazzola et al (2009)** | Chronic Pain | Group study | Patients with chronic pain: headache, fibromyalgia, neuropathic pain | Targets: past events that set the ground for pathology, present triggers of disturbance, and future adaptative response. Follow-up: no follow-up | ﻿12 weekly sessions. | Health Survey (SF-36); Anxiety (STAI), depression (BDI), personality (SCID-II), pain severity (VAS) | Pain Protocol | 38 | ﻿Reduced pain severity (VAS) (p<0·001) and improvement in all other measures. (all ps’<0·05). | 73% of patients met criteria for at least one axis II personality disorder. 44% for OCD. | .. |
| **Brennstuhl et al (2016)** | Chronic Pain | RCT  -Pilot | Patients with chronic pain. | EMDR-standard vs Non-interventional psychotherapy (control) vs EMDR-Pain  Targets: past and present triggers and future adaptative responses.  Follow-up: 1 month | 5 sessions | Post-Traumatic Checklist Scale (PCL-S), Pain Beliefs (PBPI), Multidimensional components of pain (e.g., perception, Sensorial, Cognitive) (VAS)  Follow-up= 1month. | .. | 45  (15, EMDR; 15, EMDR-Pain; 15, Control) | Standard EMDR protocol was the most effective of all, already after 5 sessions. Reduced pain perception maintained 1 month after treatment. | No group differences in post-traumatic scales. | Worsening of symptoms (PCL-S scores) in control group. |
| **Brennstuhl et al (2017)** | Chronic Pain | Case report | Patients with chronic pain (Migraine, Cervical pain, Diffuse pain) | Targets: representations of pain Follow-up: 1 month | 5 sessions | Post-Traumatic Checklist Scale (PCL-S), Pain Beliefs (PBPI). | EMDR PAIN protocol | 3 | No significant results. Tendency to decrease in all measures. | ·· | ·· |
| **Ghanbari Nia and Behnammoghadam (2018)** | Chronic Pain-Arthritis | RCT | Patients with rheumatoid arthritis | EMDR vs Guided imagery vs control.  Targets: disturbing images, and negative beliefs  Follow-up: unspecified | 6 sessions | Rheumatoid Arthritis Pain Scale (RAPS) | Standard | 75  (25 x 3groups) | Reduced pain in both interventions’ vs control (p=0·0001). Effect of EMDR was greater than guided imagery (p=0·001). | ﻿.. | ·· |
| **Höfel et al (2018)** | Chronic Pain-Arthritis | Non-RCT | Juvenile Idiopathic Arthritis (JIA) patients intolerant to Methotrexate (MTX) | Targets: not specified Follow-up: 4 months | 8 sessions over two weeks. | Intolerance severity (MISS).  Quality of Life (QoL).  Follow-up=4months. | Standard | 18 | Reduced Intolerance (p=0·001) and improved Quality of Life (p=0·008). | .. | Relapse in some patients. |
| **Willensky (2006)** | Phantom limb pain | Case report | Patients with different limbs amputation, one week to three years since amputation | Targets: accident that caused amputation Follow-up: unspecified | 3-9 sessions. | Impact of event Scale (IES), Pain disability Index (PDI), Trauma Symptom Inventory, Beck depression I and II. | Standard | 4 | Reduced pain or eliminated. | ·· | ·· |
| **Schneider et al (2008)** | Phantom limb pain | Case report | Patients with phantom limb pain | Targets: multiple events associated with the amputation, as well as a variety of targets related to issues of self-esteem, pain sensations, present triggers, and thoughts of the future. Follow-up: between 12 and 24 months | 3 to 15 sessions | Subjective distress (IES) Depression (BDI) Pain level (Faces Scale). | Standard | 5 | Reduced or eliminated phantom pain, reduced depression scores and reduced distress to subclinical levels. | .. | ·· |
| **Flik and De Roos (2010)** | Phantom limb pain | Case report | Patient with phantom limb pain | Targets: 3 vivid memories linked to the amputation Follow-up: 1 year | 10 sessions x1h | ·· | Pain protocol | 1 | Pain reduced from 10 to 1. | ·· | ·· |
| **De Roos et al (2010)** | Phantom limb pain | Group study | Patients with Chronic Phantom Limb Pain (PLP)after leg amputation | Targets: pain- related cognitions and in-session experiences.  Short term Follow-up: 3 months.  Long term Follow-up= 2.8 years. | 5 to 9 sessions | Self-evaluation of pain in a diary, Distress (SLC-90), Fatigue (CIS-20R), PTSD symptoms (IES; SIL), Quality of Life (SF-36) | Combination of EMDR standard and pain | 10 | Reduced chronic phantom limb pain-PLP (p=0·02).    Results maintained at follow-up for -ca. 50% of participants. | Overall improvement in all measures. | Tiredness, headaches during sessions and increases PLP |
| **Brennstuhl et al (2015)** | Phantom Breast Pain | Case report | Patients having undergone a mastectomy of both breasts with phantom breast syndrome | Targets: two Targets types: traumatic events related to disease experience and phantom breast sensation Follow-up: 3 & 6 months | 9 to 12 sessions (weekly, 90 min.) | Anxiety (STAI), Depression (CES) + Intensity of Pain and of Phantom Sensation | 2 Protocols where used: first the standard, and then the pain protocol | 2 | Reduced pain and Improvement of all measures. | .. | .. |
| **Olędzka et al (2016)** | Phantom - Paraparesis | Case report | Patient with paraparesis diagnosed with psychogenic pain. | Targets: Memory of exclusion from sport at school (floatback technique) Follow-up: 4 months | 6-week therapy | Depression (BDI), Pain Disability Index (PDI) | Standard | 1 | Reduced and eliminated pain. Resumed physical and social activities | Improvement of positive self-belief and confidence. | .. |
| **Sinici (2016)** | Phantom limb pain | Group study | Patients after amputation (diabetes or trauma) | EMDR one week after amputation.  Targets: picture representing pain, cognitions about effects of pain on life and personality, emotions in body and their locations Follow-up: 1 and 3 months | Between 3 and 6 sessions | Pain (MPQ), Anxiety (STAI), Depression (BDI) and the Symptom Checklist (SCL-90-R) | 8 phases, standard | 14 | Improvement in all measures (p<0·05) except anger-hostility subscale of SCL-90-R. | ·· | ·· |
| **Rostaminejad et al (2017)** | Phantom limb pain | RCT | Patients after amputation | EMDR vs control.  Targets: (a) memory of the event that caused the injury, (b) memory of the amputation, (c) memories of difficulties in functioning because of the amputation, and (d) the physical pain sensations.  Follow up: 24 months. | 12 session over one month | SUD, Pain Rating Scale | STD protocol | 60  (30, EMDR; 30, control) | EMDR was effective against PLP, positive effects maintained after 24 months (p<0·001) | ·· | ·· |
| **Brennstuhl et al (2017)** | Phantom Breast Pain | Group study | Patients with Phantom Breast Syndrome (PBS) after mastectomy | Targets: memories linked to the disease and treatment sensation of pain Follow-up: 3 and 6 months | 5 to 10 sessions | Pain and PBS sensation and intensity (numerical scale), Anxiety (STAI), Depression (CESD) + Qualitative analysis (graphic representation of pain and sensation in the breast) | Modified Grant EMDR Protocol for Pain | 8 | Reduced PBS sensation and pain. (p<0·001).  Reduced anxiety (STAI) (p<0·001) and Depression (BDI) (p<0·01).  Results maintained at 3 and 6 months. | .. | ·· |
| **Friedberg (2004)** | Fibromyalgia | Group study | Patients with fibromyalgia | Targets: focus on her most salient or intense symptom, sensation, or feeling. Follow-up: 3 months | 2 sessions | Fibromyalgia (FIQ), Fatigue (FS), Anxiety, and Depression (BDAI). Home logs of pain, stress and fatigue. Relaxation (Thermal biofeedback monitoring). | EMD Protocol | 6 | Reduced fibromyalgia impact scores (12 to 6%).  Improvement in all other measures in 4/6 subjects.  Results maintained at 3-month follow-up with further improvements on all measures | Improved relaxation (thermal biofeedback). | ﻿.. |
| **Kavakci et al (2012)** | Fibromyalgia | Group study | Patients with fibromyalgia | Targets: Memories of pain, or trauma if reported. Follow-up: no follow-up | 5 to 8 sessions | Fibromyalgia impact (FIQ), Depression (BDI), PTSD symptoms (PDS), Sleep Quality (PSQI), Anger (STAS). | ·· | 7 | Reduced tender point count (p<0·05)  Improvement in all measures (p<0·005).  Six patients no longer met FMS criteria. | .. | .. |
| **Marcus (2008)** | Migraine | Group controlled | Individuals diagnosed with classic or common migraine headache. | EMDR vs Standard care (control)  Participants treated during mid- to late-stage acute migraine and assessed at pre-treatment, posttreatment, 24 hours, 48 hours, and 7 days for migraine pain level  Targets: no targets. Follow-up: 1 day, 2 days, 1 week | 1 session x 1hour.  Diaphragmatic breathing + head compression + EMDR. | Subjective pain level (SPL), Functional assessment (MIDA), Headache Disability Inventory (HID) | Integrated EMDR (Combination of EMDR + diaphragmatic breathing + cranial compression). | 43  (21, EMDR; 22, Control) | Reduced migraine pain levels for both treatments immediately at posttreatment, 24 hours, 48 hours, and 7 days.  Greater and faster improvement with EMDR. | .. | .. |
| **Konuk et al (2011)** | Migraine | Group study | Participants with history of migraine ranging from 2 to 30 years | Targets: (a) traumatic events that were clearly connected to headaches, particularly the first experienced/ remembered headache attack, (b) re- called traumatic events that took place “relatively close in time” to the first headache attack, and (c) traumatic headache attacks (first, worst, and last) Follow-up: 3 months | Average of 8 sessions over 3 months | EMDR Headache Treatment Intake Form (developed by the authors), Weekly headache questionnaire (WHQ), SA-45 (derived from SCL-90). | EMDR Headache Protocol | 11 | Reduced headache frequency (p=0·01), average duration (p=0·050) and number of days of pain over time (p=0·027), but no reduction in pain intensity (p=0·51).  Results maintained at 3months follow up. | .. | Increase in the number of headaches during the treatment interval |
| **Hekmat et al (1994)** | Acute Pain Experimental | RCT | Volunteer participants. | EMDR vs EMDR with preferred music (EMDM) vs Control.  Interventions after iced water-noxious stimulation.  Targets: image of noxious stimulation, and beliefs related to painful image of noxious experience.  Follow-up: no follow-up | 1 session | Affect (MAACL), Pain threshold, tolerance and endurance (LS) Pain intensity (VAS), treatment credibility (5item scale). | Standard | 30  (Randomly assigned, final samples not reported) | Reduced pain (p<0·05), better tolerance (p<0·01), improvement of pain threshold (p<0·01).  Reduced anxiety (p<0·05). |  | Hypnotically susceptible participants did not respond more favourably to EMD/R. |
| **Maroufi et al (2016)** | Acute Pain  Post-Operative | RCT | Patients admitted for emergency abdominal surgery | EMDR vs Interview (Control)  Targets: descriptions of negative beliefs or images associated with the surgery.  Follow-up: no follow-up | 1 session (60min), 2 hours post-surgery | Wong-Baker FACES Pain Rating Scale | Standard | 56  (28, EMDR; 28Control) | ﻿Reduced pain intensity (p < 0·001). | ·· | .. |
| **Niraj and Niraj (2014)** | Phantom Limb | Review |  | Management of Phantom limb pain | .. | .. | .. | 3 studies | 3 studies found support the positive effects of EMDR in the treatment of PLP. | .. | .. |
| **Tesarz et al (2014)** | Chronic Pain | Review | .. | Treatment of Chronic pain | .. | .. | .. | 12 studies | Controlled trials demonstrated significant improvements in pain intensity with high effect sizes | Results maintained at follow up. No adverse events reported. | .. |
| **Tefft and Jordan (2016)** | Chronic Pain | Review | .. | Treatment of Chronic pain | .. | .. | .. | 28 studies | Overall support of positive effects of EMDR as an alternative treatment | .. | .. |
| **Wicking et al (2017)** | Chronic Pain | Review | .. | Effects of EMDR in patients with chronic pain without post-traumatic stress disorder | .. | .. | .. | 4 studies | Partial support of EMDR effectiveness. No clear results.  Lack of RCTs | .. | .. |

**Table.S7. Characteristics of studies related to Somatoform Disorders**

**Abbreviations Table 9.** TFI= Tinnitus Functional Index. Mini-TQ= Mini-Tinnitus Questionnaire. SQL-90= Symptom Checklist-90. SRIP= Self-Rating Inventory for PTSD. THI=Tinnitus handicap inventory. BDI=Beck depression inventory. BAI=Beck anxiety inventory. SF-36= Short Form Health Survey.

|  | **Topic** | **Design** | **Population** | **Treatment and Setting** | **Number of Sessions** | **Measures** | **EMDR protocol** | **Sample Size, n** | **Main Result** | **Secondary Outcome** | **Other /adverse effects** |
| --- | --- | --- | --- | --- | --- | --- | --- | --- | --- | --- | --- |
| **Rikkert et al. (2018)** | Tinnitus | Group study | Patients with chronic tinnitus. | EMDR vs waiting list (within).  Targets: negative memories and images related to negative tinnitus Follow-up: 3 months after treatment | 6 x 90 minutes sessions. | Tinnitus related distress (TFI; Mini-TQ), Distress (SCL-90) and the PTSD symptoms (SRIP). | Tinnitus EMDR | 35 | Reduced tinnitus distress (p<0·001, d= 0·72).  Results maintained at follow-up. | Reduced scores of all measures (Mini-TQ, d=0·71; SCL-90, d=0·41) | .. |
| **Phillips et al. (2019)** | Tinnitus | Group study | Patients with chronic tinnitus. | Targets: if traumatic memory linked to tinnitus or negative belief + emotion + sensation representing tinnitus experience  Follow-up: 6 months | maximum of 10 x 60 min sessions (frequency: 1 session every 1 or 2 weeks). | Subjective functional impairment (THI) Depression (BDI), anxiety (BAI). | EMDR-ad hoc tinnitus protocol. | 14 | Reduced Tinnitus Handicap (p=0·0005).  Results maintained at 6 months post-discharge. | Reduced depression scores (p=0·0098). | ... |
| **Demirci et al. (2017)** | Somatic Symptom Disorder | Group controlled | Patients with Somatic Symptom Disorder (SSD) | EMDR vs Duloxetine (SSNRI) over a 6-week course of treatment.  Targets: somatic sensations and relevant painful memories Follow up: no follow-up | 6 weekly sessions of 90 minutes EMDR versus Duloxetine 30 mg/day the first week, 60mg/day from the second week | Somatisation (SCL-90) Anxiety (BAI), Depression (BDI), Quality of life (SF-36) | EMDR -somatic symptoms protocol. | 62  (31, EMDR; 31, SSNRI) | Improvement in all measures for both groups (all ps’<0·001). Higher improvements in EMDR vs Duloxetine in all measures (all ps’<0·001) | .. | ·· |
| **Chemali et al. (2004)** | Psychogenic Seizure | Case Study | Patient with daily Psychogenic Seizures. | Targets: not specified  Follow-up: 3 months | 18 months of 1 weekly session | Occurrence of psychogenic seizures | Not specified | 1 | Reduced and eliminated seizures,  results maintained at 3mo follow up. | Events subsided shortly after start of the EMDR. | Improved intimacy. |
| **Cope et al. (2018)** | Functional Neurological Disorder | Review | .. | .. | .. | .. | .. | 3 | Four out of the five total patients were successfully treated with EMDR. | .. | .. |

**Table.S8. Characteristics of studies related to Sexual Disorders**

|  | **Topic** | **Design** | **Population** | **Treatment and Setting** | **Number of Sessions** | **Measures** | **EMDR protocol** | **Sample Size** | **Main Result** | **Secondary Outcome** | **Other /adverse effects** |
| --- | --- | --- | --- | --- | --- | --- | --- | --- | --- | --- | --- |
| **Torun (2010)** | Vaginismus | Case Study | Patients with vaginismus | Targets patient1: Past/ present disturbing memories, and prospective exposure.  Targets patient 2: physical experience of distress  Follow-up: 1 and 2 months | 3x90 minutes EMDR sessions. | Ability to have sexual intercourse | Standard, and phobia protocol. | 2 | Patients were able to have pain-free intercourse after the third session.  Results maintained after 2mo follow-up. | .. | ·· |
| **Gaboraud (2018)** | Paedophilia | Case Report | Adult male, convicted for paedophilia | Targets 1: Fear to disappoint other people Targets 2: Diverse adverse Memories of childhood Follow-up: not specified | Several Months of EMDR sessions | Self-Reported | Standard Protocol - Inverted | 1 | Reduced and eliminated paraphilias | Improvement of self-esteem. | ·· |

**Table.S9. Characteristics of studies related to Addictions**.

**Abbreviations Table 11:** AM=addiction memory. TAU=Treatment as usual. OCDS=Obsessive Compulsive Drinking Scale. PEIA=Palette of EMDR interventions in addictions. AF-EMDR=addiction focused EMDR. DeTUR= desensitization of triggers and urge reprocessing. SAS= Gambling Symptom Assessment Scale. BIS-11=Barratt impulsiveness scale. SRAD= Self rating depression Scale-Zung. SRAD=Self rating anxiety scale-Zung.

|  | **Topic** | **Design** | **Population** | **Treatment and setting** | **Number of Sessions** | **Measures** | **EMDR protocol** | **Sample Size, n** | **Main Result** | **Secondary Outcome** | **Other /adverse effects** |
| --- | --- | --- | --- | --- | --- | --- | --- | --- | --- | --- | --- |
| **Hase (2008)** | Alcohol Dependence | RCT | Patients with Chronic Alcohol Dependency | EMDR vs Treatment as Usual (control)  Targets: Memories of relapse or of intense craving. Follow-up: 1 and 6 months. | 2 sessions | Alcohol craving (OCDS) pre, post, and 1mo after treatment. | Focus on Addiction Memory (AF-EMDR) | 30  (15, EMDR; 15, Control) | 1mo: cravings reduction in EMDR group (p < 0·001). Greater reduction of cravings in TAU+EMDR vs TAU (p <0·05).  6mo: lower relapse in TAU+EMDR group vs TAU (p < 0·05) | Reduced depressive symptoms in EMDR group only. | Targets and reprocessing the AM did not lead to a destabilisation of patients. |
| **Markus (2020)** | Alcohol Dependence | RCT | Patients with Alcohol Use Disorder | EMDR vs Treatment as usual (control)  Targets: According to (PEIA), negative images associated with long-term abstinence (if present), memories associated with loss of control over drinking behaviour, coping self-efficacy undermining beliefs, trigger situations, and anticipated relapse.  Follow-up: 1 and 6 Months | 7 weekly sessions | Changes in drinking behaviour as reported by participant and biomarker indices. | Addiction focused EMDR (AF-EMDR) | 109  (55, EMDR; 54, Control) | No significant additive effect of AF-EMDR on TAU in drinking behaviour (p>0·3). | Higher number of control participants reduced alcohol consumption, Higher proportion in the EMDR group experienced less craving | Individuals in control group reported higher positive effects than EMDR group (p=0·026) |
| **Bae (2015)** | Gambling | Case Series | Inpatients with pathological gambling disorder in rehabilitation. | Patients received EMDR in addition to usual care in the unit.  Targets: trigger and urges to initiate gambling behaviours.  Follow-up: 6 months. | 3 weekly sessions for 6 weeks (total: 18 sessions) focusing on the hierarchy o | Gambling Symptom(G-SAS), impulsiveness (BIS-11), Depression (SRDA-Z), Anxiety (SRAS-Z). | DeTUR. | 4 | Self-reported gambling symptoms improved (G-SAS). Abstinence from gambling was maintained after 6mo. | Improved depression, anxiety, and impulsiveness. Self-reported satisfaction with therapy. Results maintained after 6mo. | Efficacy after short treatment time. |
| **van Minnen (2020)** | Gambling | Group Study | Patients with Gambling Disorder | AF-EMDR therapy on gambling urge and increased experienced self-control.  Targets: according to Palette of EMDR interventions in addiction (PEIA).  Follow-up: no follow-up | 1 session / week for 6 weeks AF-EMDR preceded by a 3- to 7-week non-treatment baseline phase. | Self-Report | PEIA - AF-EMDR | 8 | Three participants recovered during baseline period, two did not respond to treatment, and three improved during EMDR. | ·· | No adverse effects were noted |
| **Qurishi (2017)** | Substance Abuse | Case Study | 24yo female, Daily use of GHB and amphetamines for some years. | Targets: memory representations eliciting the strongest cravings (according to PEIA) Follow-up: 1 and 6 months. | 1 session / week for 7 weeks | Self-Report + Urine Control | PEIA | 1 | Six months following EMDR, urine control was still negative for GHB. No lapses or relapses in that period | .. | ·· |
| **Pilz (2017)** | Addictions | Review | ·· | Effects of EMDR in Substance Use Disorder. | .. | ·· | ·· | 4 studies | Reduced cravings | Improved fear, depression, emotion regulation and management and self-esteem. |  |
| **Markus (2019)** | Addictions | Review | ·· | Effects of EMDR in addiction. | .. | ·· | .. | 17 studies | Positive outcomes of EMDR in addiction. Summary of (PEIA). | .. |  |
|  |  |  |  |  |  |  |  |  |  |  |  |

**Table. S10. Characteristics of studies related to Eating Disorders**.

**Abbreviations Table 12.** ED= Eating disorders. BDI=Beck depression inventory. BAI= Beck anxiety inventory. SRT= Standard residential eating disorders treatment.

|  | **Topic** | **Design** | **Population** | **Treatment and Setting** | **Number of Sessions** | **Measures** | **EMDR protocol** | **Sample Size, n** | **Main Result** | **Secondary Outcome** | **Other /adverse effects** |
| --- | --- | --- | --- | --- | --- | --- | --- | --- | --- | --- | --- |
| **Yasar et al (2019)** | Restrictive Food Intake Disorder | Case Study | Patients with food restrictions and low weight | Target: memory related to food intake  Follow-up: not specified | 5 sessions of EMDR, then 9 sessions of CBT, then 2 sessions of EMDR | Depression (BDI) Anxiety (BAI) | .. | 2 | Reduced depression and anxiety scores  Depression:(Patient 1: 14 to 4, Patient 2: 21 to 3)  Anxiety: (Patient 1: 35 to 12 Patient 2: 35 to 5 ) | Normal Food Intake after treatment | ·· |
| **Hudson et al (1997)** | Eating Disorders | Review | ·· | .. | ·· | ·· | ·· | 1 study | No Benefit found | No theoretical basis supports EMDR effectiveness in ED. | Possibility of inducing false memories and delay evidence-based approaches |
| **Balbo et al (2017)** | Eating Disorders | Review | ·· | .. | ·· | .. | ·· | 5 studies | Beneficial effects of EMDR as a complement to standard treatment (SRT).  Only one randomized group study. | .. | .. |

**Table.S11. Characteristics of studies related to Disorders of Adult Personality.**

**Abbreviations Table 13.** IASC= Inventory of Altered Self Capacities. BPD= Borderline Personality Disorder Checklist. DES= Dissociative Experience Scale. BDI-II= Beck Depression Inventory-II-second edition. BAI= Beck anxiety inventory. AAI= Adult Attachment Interview. DeTUR= desensitization of triggers and urge reprocessing.

|  | **Topic** | **Design** | **Population** | **Treatment and Setting** | **Number of Sessions** | **Measures** | **EMDR protocol** | **Sample Size, n** | **Main Result** | **Secondary Outcome** | **Other /adverse effects** |
| --- | --- | --- | --- | --- | --- | --- | --- | --- | --- | --- | --- |
| **Brown and Shapiro (2006)** | Borderline Personality Disorder | Case Study | Patient with comorbid borderline personality disorder and major depressive disorder. | Target: disturbing memories of relationships, present triggers, imagined future actions.  Follow-up: unspecified | 20 sessions over 6 months | Functioning (IASC) | Standard | 1 | Improvement of overall functioning (IASC) | .. | .. |
| **Safarabad et al (2018)** | Borderline Personality Disorder | Case Study | Patient with borderline personality disorder | Target: Adverse Life Events.  Follow-up: 3 months | 20 sessions | Borderline symptoms (BPD) Dissociative symptoms (DES-II), Depression, (BDI-II), Anxiety (BAI) | Standard Protocol | 1 | Reduced borderline symptoms: 161 to 69 (BPD).  Results were maintained at follow-up. | Improved dissociative scores, depression and anxiety.  Results, maintained at follow-up. | .. |
| **Annesley et al (2019)** | Self-injury in personality disorder | Case Report | Patient detained within a high secure hospital with complex mental health difficulties | Target: triggers for urges to self-injure identified by patient.  Follow-up: 2 weeks, 2 months, 4 months | 18 sessions x 1hour. | Attachment status (AAI) | DeTUR EMDR | 1 | Reduced and eliminated urges to 0. | Improved mood, thinking, sleep, concentration, memory and experience of flashbacks. | .. |
| **Wesselman et al (2009)** | Attachment | Case Study | Patients with symptoms of depression and interpersonal problems | Target: recent and past difficult situations  Follow-up: not specified |  | Attachment status (AAI) | ·· | 3 | Improved attachment status and improved functioning in their relationships. | Reduced anger, shame, and fear. Improvement of self-worth, vulnerability, powerlessness, and respond rationally in their relationships. | .. |

**Table.S12. Characteristics of studies related to Neurodegenerative Disorders**.

**Abbreviations Table 14.** BDI= Beck depression inventory. HADS-A= Hospital anxiety and depression scale-A.

|  | **Topic** | **Design** | **Population** | **Treatment and Setting** | **Number of Sessions** | **Measures** | **EMDR protocol** | **Sample Size, n** | **Main Result** | **Secondary Outcome** | **Other /adverse effects** |
| --- | --- | --- | --- | --- | --- | --- | --- | --- | --- | --- | --- |
| **Amano and Toichi (2015)** | Dementia | Case study | Patients with Dementia (moderate and late stage) | Target: Behavioural and psychological symptoms of dementia (e.g., emotionality, screams).  Follow-up: no follow-up | 5-6 sessions over 3 months | Overall behaviour (e.g., Screams, restlessness, wandering, cursing, physical violence). | "on-the-spot-EMDR" standard with Tapping method. | 3 | Improvement in all measures | ·· | ·· |
| **Van Der Wielen et al (2019)** | Dementia | Case study | Patient with mild stage Alzheimer | Target: distressing memories of patients’ mother who suffered from dementia and feelings of guilt  Follow-up: 4 days, 1month, 2months, 6months, 7 months, 13 months. | One single session | Flashbacks distress (self-report), depression and anxiety (HADS; BDI). | Standard | 1 | Reduced perceived distress of emotional flashbacks.  Results maintained at follow-up.  No change in depression and anxiety scores post-intervention or at follow-up. | ·· | .. |

**Table.S13. Characteristics of studies related to Sleep Disorders**.

**Abbreviations Table 15.** ISI= Insomnia Severity Index.

|  | **Topic** | **Design** | **Population** | **Treatment and Setting** | **Number of Sessions** | **Measures** | **EMDR protocol** | **Sample Size, n** | **Main Result** | **Secondary Outcome** | **Other /adverse effects** |
| --- | --- | --- | --- | --- | --- | --- | --- | --- | --- | --- | --- |
| **Nia et al (2019)** | Insomnia | RCT | Patients with rheumatoid arthritis suffering from insomnia | EMDR vs Guided imagery, vs Control.    Target: unspecified Follow-up: mentioned but not specified | 1 daily session for 6 days | Insomnia (ISI) | Standard | 75  (25 x group) | Reduced insomnia (ISI) in EMDR (95% CI: 10·1-11·9), and Guided Imagery (95% CI: 14·4-16·2).  Both interventions were better than control (p<0·001).    Greater reduction of insomnia scores in EMDR vs Guided imagery (p=0·001). | ·· | Random sampling not possible, only random condition (Block) |

**Table.S14. Characteristics of studies related to mental disorders of Childhood and Adolescence**.

**Abbreviations Table 16.** SPQ-C = Spider Phobia Questionnaire for Children. DQ= Disgust Questionnaire. Evivo= Exposure therapy in vivo. Evirtual= computerised exposure therapy. SAM= Self-assessment Manikin for non-verbal assessment. BAT= Behaviroural avoidance test. MDD= Major depressive disorder. HDRS = Hamilton Depression Rating Scale. BDI= Beck depression inventory. VAS = visual analogue scale. ACT= asthma control test. PAQLQ(S)= Pediatric Quality of Life Questionnaire. CBCL= Child Behavior Checklist. YSR= Youth Self Report. YGTSS= Yale Global Tic Severity Scale. CY-BOCS= Child Yale Brown Obsession and Compulsion Scale. SAFA = Self-administered Scales for Children and Adolescents.

|  | **Topic** | **Design** | **Population** | **Treatment and Setting** | **Number of Sessions** | **Measures** | **EMDR protocol** | **Sample Size, n** | **Main Result** | **Secondary Outcome** | **Other /adverse effects** |
| --- | --- | --- | --- | --- | --- | --- | --- | --- | --- | --- | --- |
| **De Jong et al (1997)** | Anxiety disorders  -Phobia | Group Study | Patients with arachnophobia | Target: (a) the most aversive confrontation with spiders, (b) the most recent aversive confrontation with spiders, and (c) a future confrontation with spiders  Follow-up: no follow-up | 1 session x 1.5h. + exposure in vivo 1.5h.  On phobic children only | Spider fear (SPQ-C),  Disgust sensitivity (DQ), Spider disgust (DQ-spider). | EMDR for phobias | 114  (21, Phobic children /14fathers, 17mothers), 22, non-phobic children /19fathers, 21mothers) | Reduced spider fear (SPQ-C) after treatment (p<0·05).  Reduced spider disgust (DQ-spider) after treatment (p<0·05). | Improvement of spider disgust and fear are correlated (r=0·43, p<0·05). | Parents of phobic children are more spider fearful vs. parents of non-phobic children (p<0·05). All mothers find spiders more disgusting vs fathers (p=0·05). Acquisition of spider fear may be facilitated by parental reactions. |
| **Muris et al (1998)** | Anxiety disorders  -Phobia | Group controlled | Patients with arachnophobia | Phase 1 (T1): EMDR vs, Exposure in vivo (Evivo), vs virtual exposure (Evirtual).  Phase 2 (T2): Exposure in vivo for all.  Targets: Past, recent and prospective most aversive confrontation with spiders  Follow-up**:** no follow-up | 1 session x 2.5h (Phase 1)  1 session x 1.5h (Phase2) | Subjective fear (SPQ)-C; SAM Behavioural avoidance (BAT)  Subjective effectiveness of treatment (Likert scale).  Time: T0, T1, T2. | EMDR for phobias | 26  (9, EMDR; 9, Evivo; 8, Evirtual). | Reduced fear (SAM) after EMDR (p<0·005) only at T1(<T0).  Reduced fear (SPQ; SAM) after Evivo only at T1 (<T0).  Improved behavioural avoidance (BAT) for EMDR group only after T2 (p=0·008); Evivo after T1 (p<0·001).  Improvement of all measures greater for Evivo group vs EMDR (all ps’<0·05). | Reduced anxiety (BAT) in Evivo group only (p<0·005). | Evivo positive effects were faster; remains the treatment of choice for children. |
| **Bae et al. (2008)** | Mood disorders | Case report | Patients with major depressive disorder (MDD) and no history of trauma | Target: stressful memories and interpersonal/relational issues.  Follow-up: 2 and 3 months | 3 and 7 sessions | Depression (HDRS; BDI) | Standard | 2 | Reduced depressive symptoms and complete remission, Results maintained at 2 and 3mo follow-up. | ·· | .. |
| **Verkleij et al (2017)** | Anxiety disorders  -Asthma | Case Report | Patient with difficult to control asthma | CBT first + EMDR at end phase.  Target: memories specific to traumatic asthma exacerbations.  Follow-up: 1 and 2 weeks | 13 sessions CBT, 3 sessions EMDR. 1h x sessions week. | Asthma exacerbations, physical and social activities, physical complaints, worrying (VAS).  Control of asthma (ACT), Quality of Life (PAQLQ(S)), Emotion and behaviour (CBCL= YSR), lung function. | Children’s protocol | 1 | Improvement of all measures (VAS) except physical complaints after therapy.  Improvement on all measures was achieved at follow-up. | Improvement in all domains (ACT, PAQLQ, CBCL, YSR) as from post-treatment. | .. |
| **Guido et al (2019)** | Autoimmune disorders | Case Report | Patient with Pediatric Autoimmune Neuropsychiatric Disorder associated with streptococcus (PANDAS) | Target: memories of acute episodes related to tics and seizures.  Follow-up: 6months follow-up | 8 sessions (preceded by training sessions with the parents) | Tic Severity (YGTSS) and Obsessive-Compulsive symptoms (CY-BOCS). | Standard | 1 | Reduced tic severity (YGTSS) score from moderate to none.  Reduced obsessive compulsive symptoms (CY-BOCS), score from moderate to minimal. | ·· | ·· |
| **Mariani Wigley et al (2019)** | Anxiety disorders  -surgical procedures | RCT | Pediatric patients undergoing painful and invasive medical procedures | EMDR vs Nonpharmacological Technique (NPT) group.  Target: unknown. Follow-up**:** no follow-up | 1 session | Anxiety and depression (SAFA) | Standard | 49  (24, EMDR; 25, NPT) | Reduced anxiety (SAFA) in EMDR vs NPT (p=0·038) | .. | ·· |

| Table S.15. Distribution of Protocol variations across disorders categories | | | | | | | | | | | | | | |
| --- | --- | --- | --- | --- | --- | --- | --- | --- | --- | --- | --- | --- | --- | --- |
| **PROTOCOL** | **MOOD** | **STRESS** | **ANXIETY** | **PERF_ANXIETY** | **OCD** | **PAIN** | **SOMATOFORM** | **SEXUAL** | **ADDICTIONS** | **EATING** | **ADULT_PERS** | **NEURODEG** | **SLEEP** | **CHILDHOOD/ADOLESCENCE** |
| AF-EMDR |  |  |  |  |  |  |  |  |  |  |  |  |  |  |
| Children Protocol |  |  |  |  |  |  |  |  |  |  |  |  |  |  |
| DeprEnd |  |  |  |  |  |  |  |  |  |  |  |  |  |  |
| DeTUR |  |  |  |  |  |  |  |  |  |  |  |  |  |  |
| Oncology symptoms |  |  |  |  |  |  |  |  |  |  |  |  |  |  |
| Headache symptoms |  |  |  |  |  |  |  |  |  |  |  |  |  |  |
| Integrated_EMDR |  |  |  |  |  |  |  |  |  |  |  |  |  |  |
| Leeds_Model |  |  |  |  |  |  |  |  |  |  |  |  |  |  |
| EMDR_OCD_Pozza |  |  |  |  |  |  |  |  |  |  |  |  |  |  |
| Pain |  |  |  |  |  |  |  |  |  |  |  |  |  |  |
| Pain_ModGrant |  |  |  |  |  |  |  |  |  |  |  |  |  |  |
| Pain+Standard |  |  |  |  |  |  |  |  |  |  |  |  |  |  |
| PEIA |  |  |  |  |  |  |  |  |  |  |  |  |  |  |
| Phobia protocol |  |  |  |  |  |  |  |  |  |  |  |  |  |  |
| Phobia_adapted |  |  |  |  |  |  |  |  |  |  |  |  |  |  |
| Phobia+Standard |  |  |  |  |  |  |  |  |  |  |  |  |  |  |
| Somatic symptons |  |  |  |  |  |  |  |  |  |  |  |  |  |  |
| Standard |  |  |  |  |  |  |  |  |  |  |  |  |  |  |
| Standard - Inverted |  |  |  |  |  |  |  |  |  |  |  |  |  |  |
| Standard_Tapping |  |  |  |  |  |  |  |  |  |  |  |  |  |  |
| Tinnitus symptoms |  |  |  |  |  |  |  |  |  |  |  |  |  |  |
| Unspecified/ no data |  |  |  |  |  |  |  |  |  |  |  |  |  |  |
| Wingwave |  |  |  |  |  |  |  |  |  |  |  |  |  |  |
| *Different variations or types of EMDR protocols were found. AF_EMDR= Addiction Focused EMDR (Markus et al., 2020); | | | | | | | | | | | | | | |
| DeTUR= Desensitization of triggers and urge reprocessing (Popky, 2005); DeprEnd= Depressive disorders protocol (Hofmann et al., 2016); | | | | | | | | | | | | | | |
| Integrated _EMDR= EMDR + diaphragmatic Breathing + cranial compressions; Leeds_Model= Leeds model for panic disorders (Leeds, 2009;2012); | | | | | | | | | | | | | | |
| EMDR_OCD_Pozza= Protocol for resistant obsessive-compulsive disorder (Pozza et al., 2014); Pain= Pain protocol (Grant, 1999); Pain_ModGrant=Modified Grant protocol; | | | | | | | | | | | | | | |
| Pain+Standard = combination of pain + standard protocol; Standard = standard protocol developped by Shapiro (1995); Standard_Phase4= Only phase 4 was administered. | | | | | | | | | | | | | | |
| Standard_tapping = standard protocol with finger tapping; | | | | | | | | | | | | | | |
| PEIA = palette of EMDR interventions in addiction (Markus & Hornsveld, 2017); Phobia protocol= standard phobia protocol (Shapiro 2001); | | | | | | | | | | | | | | |
| Phobia_adapted = present targets are processed first, past targets last (Marr, 2012); Wingwave = standard EMDR + Bi-Digital-O-Ring-Test (BDORT) (Besser-Siegmund and Siegmund 2010, 2013) | | | | | | | | | | | | | | |

**Table.S15. Overview of protocol variations across disorder categories**.

**Table.S16 and S17. Distribution of studies per category and protocol variation.**

| Table.S16 Studies by Classification and Intervention Protocol | | | | | |
| --- | --- | --- | --- | --- | --- |
|  |  |  |  |  |  |
| **MOOD** | **STRESS** | **ANXIETY** | **PERFORMANCE ANXIETY** | **OCD** | **SOMATOFORM** |
| ***DeprEnd*** | ***EMDR Oncology*** | ***Leeds_Model*** | ***Wingwave*** | ***NA*** | ***NA*** |
| Ostacoli et al (2018) | Dinapoli (2019) | Bhagwagar (2016) | Rathschlag and Memmert (2014) | Corrigan and Jennett (2004) | Chemali et al. (2004) |
| ***Standard*** | ***NA*** | ***Phobia*** | ***Standard*** | ***OCD _Pozza2014*** | ***SomaticSymptons*** |
| Behnammoghadam et al (2015) | Zolghadr (2019) | de Jongh (2012) | Aslani et al (2014) | Mazzoni et al (2017) | Demirci et al. (2017) |
| Fereidouni et al (2019) | ***Standard*** | Lapsekili and Yelboga (2014) | Brooker (2018) | ***Phobia_adapted*** | ***Tinnitus*** |
| Gauhar (2016) | Borji (2019) | Muris and Merckelbach (1995) | Carrigan and Levis (1999) | Marr  (2012) | Phillips et al. (2019) |
| Guina and Guina (2018) | Hatefi (2019) | ***Standard*** | Falls et al (2018) | ***Phobia*** | Rikkert et al. (2018) |
| Hase et al(2015) | Szpringer (2018) | Faretta (2012) | Foley and Spates (1995) | Marsden (2016) |  |
| Hofmann et al (2014) | Trznadel (2017) | Farima et al (2015) | Maxfield and Melnyk (2000) | Marsden et al (2018) |  |
| Jahanfar et al (2020) | Standard_Phase4 | Fernandez and Faretta (2007) | Vauthier et al (2019) | ***Standard*** |  |
| Minelli et al (2019) | Rahimi (2019) | Goldstein and Feske (1994) |  | Bohm and Voderholzer (2010) |  |
| Rosas Uribe et al(2010) |  | Goldstein et al (2000) |  | Nazari et al (2011) |  |
|  |  | Nicolas and Vautier (2017) |  |  |  |
|  |  | Sagaltici and Demirci (2019) |  |  |  |
|  |  |  |  |  |  |

| Table.S17 Studies by Classification and Intervention Protocol | | | | |
| --- | --- | --- | --- | --- |
|  |  |  |  |  |
| **PAIN** | **SEXUAL** | **ADDICTIONS** | **EATING** | **PERSONALITY** |
| ***Headache*** | ***PhobiaSTD*** | ***DeTUR*** | ***NA*** | ***DeTUR*** |
| Konuk et al (2011) | Torun (2010) | Bae (2015) | Yazar et al (2019) | Annesley et al (2019) |
| IntegratedEMDR | **Standard Protocol - Inverted** | ***PEIA*** |  | ***NA*** |
| Marcus (2008) | Gaboraud (2018) | Qurishi (2017) |  | Wesselman et al (2009) |
| ***NA*** |  | van Minnen (2020) |  | ***Standard*** |
| Kavakci et al (2012) |  | ***AF-EMDR*** |  | Brown and Shapiro (2006) |
| ***Pain*** |  | Hase (2008) |  | Safarabad et al (2018) |
| Brennstuhl et al (2016) |  | Markus (2020) |  |  |
| Brennstuhl et al (2017) | **NEURODEGENERATIVE** | **SLEEP** | **CHILDHOOD & ADOLESCENSE** | |
| Flik and De Roos (2010) | ***Standard*** | ***Standard*** | ***Children*** | |
| Mazzola et al (2009) | Amano and Toichi (2015) | Nia et al (2019) | Verkleij et al (2017) | |
| ***PainGrant*** | ***Standard_Tapping*** |  | ***Phobia*** | |
| Brennstuhl et al (2017) | Van Der Wielen et al (2019) |  | De Jong et al (1997) | |
| ***PainSTD*** |  |  | Muris et al (1998) | |
| Brennstuhl et al (2015) |  |  | ***Standard*** | |
| De Roos et al (2010) |  |  | Bae et al. (2008) | |
| Grant and Threlfo (2002) |  |  | Guido et al (2019) | |
| ***Standard*** |  |  | Mariani Wigley et al (2019) | |
| Friedberg (2004) |  |  |  |  |
| Ghanbari Nia and Behnammoghadam (2018) |  |  |  |  |
| Hekmat et al (1994) |  |  |  |  |
| Höfel et al (2018) |  |  |  |  |
| Maroufi et al (2016) |  |  |  |  |
| Olędzka et al (2016) |  |  |  |  |
| Ray and Page (2002) |  |  |  |  |
| Rostaminejad et al (2017) |  |  |  |  |
| Schneider et al (2008) |  |  |  |  |
| Sinici (2016) |  |  |  |  |
| Brennstuhl et al (2016) |  |  |  |  |
| Willensky (2006) |  |  |  |  |

| Table.S18. Number of studies reporting clear Success, Intermediate success and no improvement after EMDR treatment. | | | |
| --- | --- | --- | --- |
|  |  |  |  |
| **Study Type** | **SUCCESS** | **INTERMEDIATE** | **No-Improvement** |
| RTC | 20 | 3 | 4 |
| Control Group | 7 | 3 |  |
| Group Study | 11 | 1 |  |
| Case study/Reports | 38 | 2 | 1 |
|  |  |  |  |
| **Group** |  |  |  |
| PAIN | 20 |  | 2 |
| ANXIETY | 9 | 2 |  |
| MOOD | 8 | 2 |  |
| STRESS | 7 |  |  |
| PERFORMANCE ANXIETY | 6 | 1 | 1 |
| CHILDHOOD & ADOLESCENSE | 5 | 1 |  |
| OCD | 4 | 2 | 1 |
| PERSONALITY | 4 |  |  |
| SOMATOFORM | 4 |  |  |
| ADDICTIONS | 3 | 1 | 1 |
| NEURODEGENERATIVE | 2 |  |  |
| SEXUAL | 2 |  |  |
| EATING | 1 |  |  |
| SLEEP | 1 |  |  |

**Table.S18. Distribution of number of studies that reported success, intermediate success and no improvement.**

**Quality of Evidence Assessment: Guidelines for Quality Assessment are partially based on the GRADE system (Atkins et al., 2004).**

Building upon the ad-hoc GRADE system to assess the quality of the evidence of the studies, a series of criteria, primarily methodological, are assessed and operationalised as increasing or decreasing in values, according to the positive or negative nature and magnitude of the assessment (e.g., High, Moderate, low levels of inconsistency, or strength of design).  The assessment was performed independently by the authors allowing an appraisal of the quality of the evidence of the group studies.

Our quality assessment considers different components for qualitative grading related to (a)The Design, (b) The Patients, (c) The interventions, (d) The comparisons made, and (e)The outcomes.

Here next, a list of criteria considered:

- Study Design: (e.g., Strong (RCT), Moderate (controlled group), weak (group))
- Study methodological limitations:

1. Blinding (control for Observation bias) = Not applicable. All studies have observation bias. Patients and Clinicians/Researchers know what treatment they will receive.
2. Allocation concealment (control for Selection Bias): Not applicable in the case of group studies and RCT.
3. Type of control:

- Control level: Grade = number of control groups.
- Control Type: CBT/“Gold Standard”/TAU =Strong control, Waiting list=Moderate, No treatment= very low control.
- Outcome Measures:

1. Main outcome: Ad-hoc measure (weak measures) vs. Standardised measures (Strong measures). (e.g., Did the authors used a liberal scale to assess recovery or did they use a specific standard scale/questionnaire/battery/psychometric tool to measure the reduction of migraines? Depression? Etc).
2. Secondary outcomes (if applicable): Ad-hoc vs Standardised (same as above).
3. Baselines/ Multiple baselines: Stronger evidence if multiple baselines (e.g., Did the study consider any baseline measurements? Any multiple baselines?)
4. Failure to report outcomes/No effect. (Fail=weak; Success= moderate; Better than control =strong).

- Inconsistency of results:

1. Heterogenous results (e.g., Not better than control/Not better than other therapy/Paradoxical results of control or patients). If YES = High level of inconsistency.
2. Heterogenous interventions: (e.g., was EMDR treatment given alone (Strong evidence), or was EMDR mixed or part of a simultaneous therapy (Strong limitation)?).

- Maintenance-consistency of effects: Positive results Maintained at Follow-Up (High consistency); Reduced effects at Follow-up (Weak consistency).

- Imprecision:

1. Small sample size? Small SZ (<30) vs. Sufficient (>30) =2. (Following the Central Limit Theorem).
   - Outcomes:
2. Critical/main outcomes and Secondary outcomes: Strong outcomes (success in both measures), Moderate (Success in only the Critical/main outcomes), Weak (only secondary outcomes successful).
